# Cross-Talk between NFkB and the PI3-Kinase/AKT Pathway Can Be Targeted in Primary Effusion Lymphoma (PEL) Cell Lines for Efficient Apoptosis

**DOI:** 10.1371/journal.pone.0039945

**Published:** 2012-06-29

**Authors:** Azhar R. Hussain, Saeeda O. Ahmed, Maqbool Ahmed, Omar S. Khan, Sally Al AbdulMohsen, Leonidas C. Platanias, Khawla S. Al-Kuraya, Shahab Uddin

**Affiliations:** 1 Human Cancer Genomic Research, Research Center, King Faisal Specialist Hospital and Research Center, Riyadh, Saudi Arabia; 2 Feinberg School of Medicine, Robert H. Lurie Comprehensive Cancer Center, Northwestern University, Chicago, Illinois, United States of America; University of Pecs Medical School, Hungary

## Abstract

**Background:**

A number of constitutively activated signaling pathways play critical roles in the survival and growth of primary effusion lymphoma cells (PELs) including NFkB and PI3/AKT kinase cascades. NFkBis constitutively activated in a number of malignancies, including multiple myeloma, Burkitt’s lymphoma and diffuse large cell B-cell lymphoma. However, its role in primary effusion lymphoma has not been fully explored.

**Methodology/Principal Findings:**

We used pharmacological inhibition and gene silencing to define the role of NFkB in growth and survival of PEL cells. Inhibition of NFkB activity by Bay11-7085 resulted in decreased expression of p65 in the nuclear compartment as detected by EMSA assays. In addition, Bay11-7085 treatment caused de-phosphorylation of AKT and its downstream targets suggesting a cross-talk between NFkB and the PI3-kinase/AKT pathway. Importantly, treatment of PEL cells with Bay11-7085 led to inhibition of cell viability and induced apoptosis in a dose dependent manner. Similar apoptotic effects were found when p65 was knocked down using specific small interference RNA. Finally, co-treatment of PEL cells with suboptimal doses of Bay11-7085 and LY294002 led to synergistic apoptotic responses in PEL cells.

**Conclusion/Significance:**

These data support a strong biological-link between NFkB and the PI3-kinase/AKT pathway in the modulation of anti-apoptotic effects in PEL cells. Synergistic targeting of these pathways using NFKB- and PI3-kinase/AKT- inhibitors may have a therapeutic potential for the treatment of PEL and possibly other malignancies with constitutive activation of these pathways.

## Introduction

Human infection by KSHV/HHV-8 is associated with the development of at least three proliferative disorders: Kaposi’s sarcoma (KS), primary effusion lymphoma (PEL) and a subset of multicentric Castleman’s disease (MCD) [1]. Primary effusion lymphoma (PEL) is a variant of non-Hodgkin’s lymphoma that is mainly infected by Kaposi sarcoma associated herpesvirus (KSHV) and sometimes also co-infected with Epstein - Barr virus (EBV) [2]. There are reports demonstrating that PEL can occasionally occur in HIV-negative patients, especially in organ transplant recipients and in patients with chronic hepatitis B [3], [4], [5], [6]. Morphologically, PEL shares features of large-cell immunoblastic and anaplastic large-cell lymphoma [3], [7]. Pleural and abdominal effusions from patients with PEL contain a number of cytokines, which serve as autocrine growth factors [8]. For example, IL-10 has been reported to serve as autocrine growth factor for AIDS-related B-cell lymphoma [9], while it has also been shown that PEL cells use viral IL-6 and IL-10 in an autocrine fashion for their survival and proliferation [8], [9].

A number of constitutively activated signaling pathways play critical roles in the survival and growth of PEL cells [10]. These include NFkB, PI3-kinase/AKT and JAK/STAT survival pathways [11], [12], [13]. NFkB is now widely recognized as a key positive regulator of cancer cell proliferation and survival via its ability to transcriptionally activate many pro-survival and anti-apoptotic genes such as XIAP, Bcl-2, Bcl-Xl, IκB-α, cIAP1, cIAP-2 and survivin [14]. NFkB is a family of 5 transcriptional factors including p50, p52, p65 (Rel-A), RelB and c-Rel, all of which contain a REL homology domain (RHD) at the N-terminus which mediates their dimerization, nuclear localization and DNA binding [15].

A number of dysregulated survival pathways have the ability to cross-talk with other survival pathways thereby increasing the aggressiveness of various cancers [16], [17]. Such cross-talking allows cancer cells to escape death in response to different pro-apoptotic signals, ultimately resulting in unregulated proliferation and and the emergence of more aggressive and drug-resistant phenotypes [17]. The NFκB survival pathway also has the ability to cross-talk with other survival pathways including PI3-kinase/AKT [18], [19] in various cancers. Therefore, targeting the NFκB pathway alone may not be sufficient to induce apoptosis of malignant cells and combinations of various inhibitors maybe required to achieve the desired effect.

Apoptosis is required for the normal homeostasis of normal cells [20] and is associated with specific cellular features, such as shrinkage, nuclear blebbing, chromatin condensation and fragmentation of DNA [21]. There are two major pathways by which apoptosis can be initiated; extrinsic or death receptor pathway or intrinsic or mitochondrial pathway [22]. Even though, the two pathways may act independently of each other, they converge at the level ofcaspase-3. Both the apoptotic pathways also have the ability to cross-talk at the level of caspase-8. Although both these apoptotic pathways can induce apoptosis alone for efficient apoptosis to occur, both pathways need to be activated simultaneously.

In this study, we first examined the constitutive activity of NFκB in PEL cell lines followed by inhibition of NFκB activity by the specific inhibitor, Bay11-7085, in order to define the functional relevance of this pathway in PEL cells. Our data provide evidence that NFκB survival pathway is constitutively activated in PEL cells and that inhibition of this pathway negatively regulates down-stream anti-apoptotic and pro-survival targets of p65 subunit of NFκB. We also determined whether inhibition of NFκB activity would induce apoptosis in PEL cell lines. Finally, we determined whether there is a cross-talk between the NFκB pathway and the PI3-kinase/AKT pathway in PEL cells and whether combined targeting of these pathways with sub-optimal doses of inhibitors would induce a more potent apoptosis in PEL cells.

## Materials and Methods

### Cell Culture and Treatment

The human PEL cell lines BC-1, BC-3, BCBL-1 and HBL-6 were obtained from American Type Culture Collection (Rockville MD, USA) and cultured in RPMI 1640 medium supplemented with 10% (v/v) Fetal bovine serum (FBS), 100 U/ml penicillin, 100 U/ml streptomycin at 37°C in an humidified atmosphere containing 5% CO_2._ BC1 and HBL-6 cell lines are co-infected with EBV and HHV-8 while BC3 and BCBL1 cell lines are HHV-8 positive only. The cells were treated with various doses of Bay11-7085 dissolved in Dimethyl Sulfoxide (DMSO) for different time periods in RPMI culture media supplemented with 5% (v/v) FBS. The control cells were incubated with maximum used amount of DMSO only. Each experiment was performed at least three times to confirm the results.

**Figure 1:**
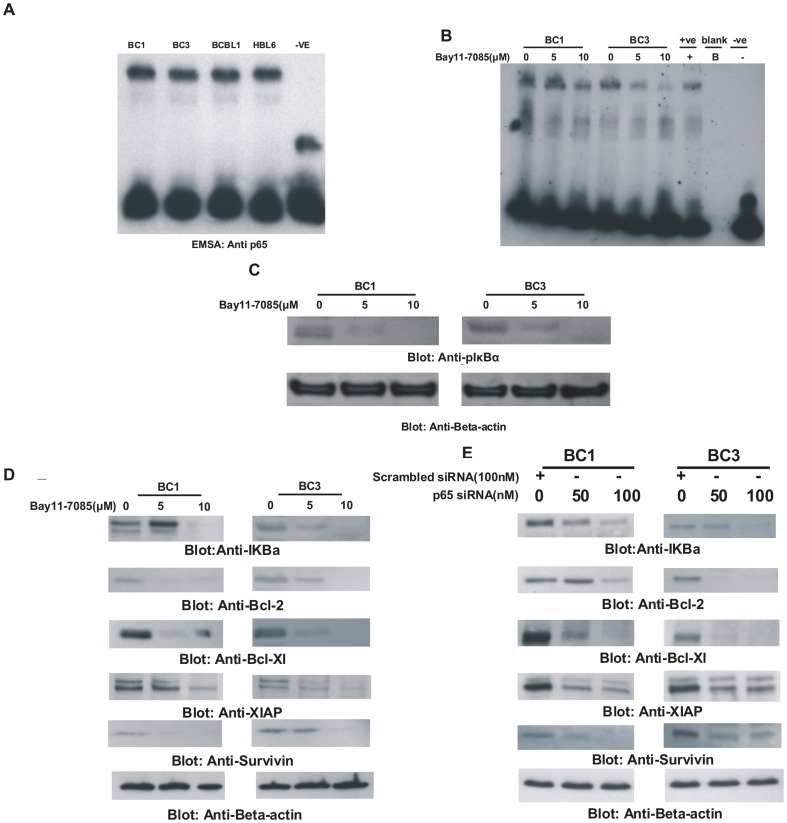
Role of NFκB in PEL cell lines (A) Constitutive expression of NFkB in PEL cells. Nuclear extracts from BC1, BC3, BCBL1 and HBL6 cell lines were prepared as described in material and Methods and electrophoretic mobility shift assay (EMSA) was performed as described in Materials and Methods. Briefly, 5×10^6^ cells were washed with cold PBS and suspended in 0.4 mL hypotonic lysis buffer containing protease inhibitors for 30 minutes. The cells were then lysed with 10% Nonidet P-40. (**B**) **Bay11-7085 inhibits constitutive nuclear NFkB in PEL cells.** BC1 and BC3 cells were treated with 5 and 10 µM Bay11-7085 for 24 hours. Nuclear extracts were prepared and EMSA was performed. (**C**) **Effect of Bay11-7085 on IκBα phosphorylation in PEL cells.** BC1 cells were treated with 5 and 10 µM Bay11-7085 for 24 hours. Cells were lysed and equal amounts of proteins were separated by SDS-PAGE, transferred to PVDF membrane, and immunoblotted with antibodies against phospho-IκBα and Beta actin as indicated. (**D**) **Bay11-7085 treatment causes down-regulation of expression of down-stream targets of p65.** BC1 and BC3 cells were treated with 5 and 10 µM Bay11-7085 for 24 hours. Cells were lysed and equal amounts of proteins were separated by SDS-PAGE, transferred to PVDF membrane, and immunoblotted with antibodies against IκBα, Bcl-2, Bcl-Xl, XIAP, Survivin and Beta-actin. (**E**) **Transcriptional down-regulation of p65 causes decreased expression of p65 targets in PEL cells.** BC1 and BC3 cells were transfected with siRNA against p65 for 48 hours. Following transfection, cells were lysed and equal amounts of proteins were separated by SDS-PAGE, transferred to PVDF membrane, and immunoblotted with antibodies against IκBα, Bcl-2, Bcl-Xl, XIAP, Survivin and Beta-actin.

### Reagents and Antibodies

Bay11-7085 was purchased from Tocris (Ellisville, MI). Bax 6A7 monoclonal antibody and DMSO were purchased from Sigma Chemical Co (St. Louis, MO). zVAD-fmk and LY294002 were purchased from Calbiochem (San Diego, CA). Antibodies against p-AKT (Ser473), p-GSK3α/β (Ser21/9), p-Foxo1(Thr24/32), p-Bad (ser136), XIAP (cat:2042), Bcl-Xl (cat:2762), caspase-3 (cat:9665), caspase-9 (cat:9508), PARP-1 (cat:9542), cleaved caspase-3 (cat:9661) and BID (cat:2006) antibodies were purchased from Cell Signaling Technologies (Beverly, MA). p-IκBα (Ser32), p65 (, IκBα (C-21), cytochrome c (A-8), SKP2 (H-435), p27Kip1 (C-19), and Bax (N-20) antibodies were purchased from Santa Cruz Biotechnology, Inc. (Santa Cruz, CA). cIAP1 (AF-8171), cIAP2 (AF-8181), survivin (AF-886), and caspase-8 (clone 84131) antibodies were purchased from R&D (Minneapolis MN). Bcl-2 (clone 124) antibody was purchased from Dako (Carpinteria, CA). Annexin-FITC was purchased from Molecular Probes (Eugene OR). Apoptotic DNA-ladder kit was obtained from Roche (Penzberg, Germany). LightShift Chemiluminescent EMSA kit was purchased from Thermo Scientific (Rockford IL).

**Figure 2:**
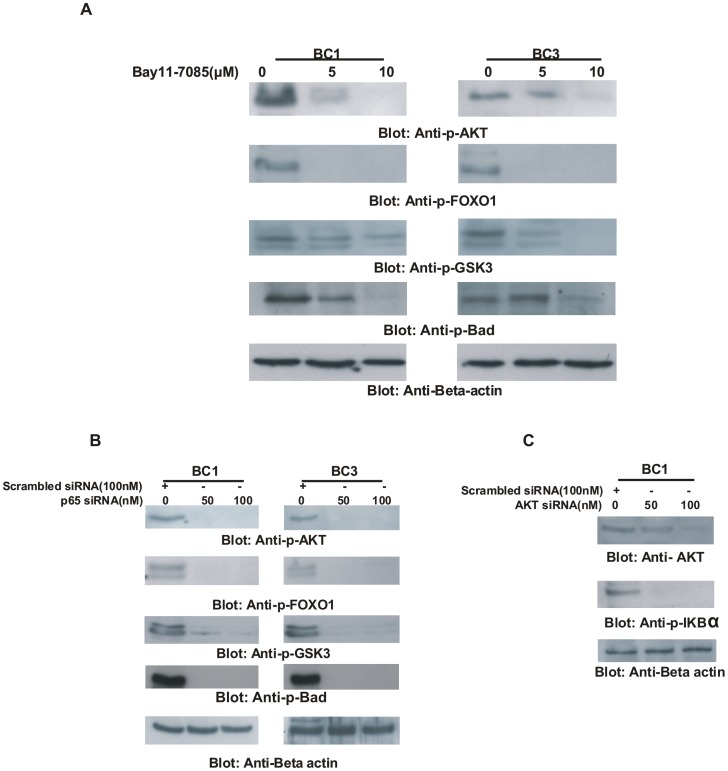
Cross-talk between NFκB and PI3-kinase/AKT pathway in PEL cell lines. (**A**) **Bay11-7085 treatment inactivates AKT and its down-stream targets in PEL cells.** BC1 and BC3 cells were treated with 5 and 10 µM Bay11-7085 for 24 hours. Cells were lysed and equal amounts of proteins were separated by SDS-PAGE, transferred to PVDF membrane, and immunoblotted with antibodies against p-AKT, p-FOXO1, p-GSK3, p-Bad and Beta-actin. (**B**) **Transcriptional knock down of p65 causes in-activation of AKT and its down-stream targets in PEL cells.** BC1 and BC3 cells were transfected with siRNA against p65 for 48 hours. Following transfection, cells were lysed and equal amounts of proteins were separated by SDS-PAGE, transferred to PVDF membrane, and immunoblotted with antibodies against p-AKT, p-FOXO1, p-GSK3, p-Bad and Beta-actin. (**C**) **Transcriptional targeting of AKT causes in-activation of NFκB pathway.** BC1 cells were transfected with siRNA against AKT for 48 hours. Following transfection, cells were lysed and equal amounts of proteins were separated by SDS-PAGE, transferred to PVDF membrane, and immunoblotted with antibodies against p-AKT, p-IκBα and Beta-actin.

### Assessment of Cell Viability by 3-(4,5-Dimethylthiazol-2-yl)-2,5-Diphenyltetrazolium Bromide (MTT) Assays

Initially, PEL cells were seeded at the concentration of 10^4^ cells in triplicates in a 96 well format. Cells were then treated with various doses of Bay11-7085 for 48 hours in a final volume of 0.2 ml for 48 hours. Cell viability was measured by MTT cell viability assay, as previously described [23], [24]. 6 wells for each dosage including vehicle control were analyzed for each experiment. * denotes statistical significance.

### Live Dead Assay

To measure apoptosis, Live-Dead assay (Invitrogen, Eugene, OR) was used as described previously [25]. Briefly, 1×10^6^ PEL cells were treated with various doses of Bay11-7085 for 24 hours. Following incubation, cells were re-suspended in 1 ml of PBS containing 50 µM calcein AM and 8 µM ethidium homodimer and cells were incubated in the dark for 20 minutes. 50 µl of suspension was placed on slides and visualized under an Olympus fluorescent microscope using a longpass filter.

**Figure 3:**
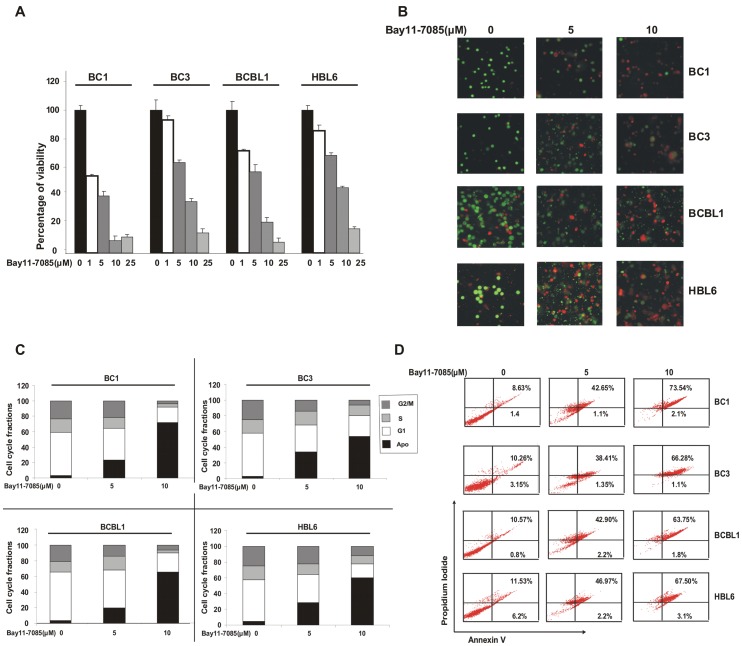
Down-regulation of NFκB pathway leads to inhibition of cell growth and induces apoptosis in PEL cell lines. (**A**) **Bay11-7085 suppresses growth of PEL cells.** PEL cell lines were incubated with 0–25 µM Bay11-7085 for 24 hours. Cell viability was measured by MTT assays as described in Materials and Methods. The graph displays the mean +/− SD (standard deviation) of three independent experiments, * p<0.05, statistically significant (Students *t*-test). **Bay11-7085 treatment induces apoptosis in PEL cells** (**B**) PEL cells were treated with 5 and 10 µM Bay11-7085 for 24 hours and apoptosis was measured by Live/Dead Assay. (**C**) PEL cells were treated with 5 and 10 µM Bay11-7085 for 24 hours. Thereafter, the cells were washed, fixed and stained with propidium iodide, and analyzed for DNA content by flow cytometry as described in “Materials and methods”. (**D**) PEL cells were treated with 5 and 10 µM Bay11-7085 (as indicated) for 24 hours and cells were subsequently stained with flourescein-conjugated annexin-V and propidium iodide (PI) and analyzed by flow cytometry.

### Cell Cycle Analysis, Annexin V Staining, and DNA Laddering

PEL cell lines were treated with different concentrations of Bay11-7085 as described in the legends. For cell cycle analysis, cells were washed once with PBS and re-suspended in 500 µl hypotonic staining buffer and analyzed by flow cytometry as described previously [26]. For detection of apoptosis, cells were harvested and percentage apoptosis was measured by flow cytometry after staining with fluorescein**–**conjugated annexin-V and propidium iodide (PI) (Molecular probes, Eugene, OR) and DNA laddering using a 1.5% agarose gel as described previously [27].

### Cell Lysis and Immunoblotting

Cells were treated with Bay11-7085 as described in the legends and lysed as previously described [28]. Briefly, and supernatant was collected. Briefly, Following treatment, cell pellets were resuspended in phosphorylation lysis buffer (0.5–1.0% Triton X-100, 150 mM NaCl, 1 mM EDTA, 200 µM sodium orthovanadate, 10 mM sodium pyrophosphate, 100 mM sodium fluoride, 1.5 mM magnesium chloride) along with protease inhibitor coctail tablet (cat 1169748001, Roche, Indianapolis IN) and incubated on ice for 1 hour, spun for 15 minutes at 14000RPM at 4°C and supernatant was collected.Protein concentrations were assessed by Bradford assay before loading the samples. 10–15 µg of proteins were separated by SDS-PAGE and transferred to polyvinylidene difluoride membrane (PVDF) (Immobilion, Millipore). Proteins were immunoblotted with different antibodies and visualized by the enhanced chemiluminescence (Amersham, Piscataway, NJ) method.

### Preparation of Nuclear Extracts for NF-kB

Nuclear extracts were prepared according to earlier study [27]. Briefly, 5×10^6^ cells were washed with cold PBS and suspended in 0.4 mL hypotonic lysis buffer containing protease inhibitors for 30 minutes. The cells were then lysed with 10% Nonidet P-40. The homogenate was centrifuged, and supernatant containing the cytoplasmic extracts was stored frozen at −80°C. The nuclear pellet was re-suspended in 25 µL ice-cold nuclear extraction buffer. After 30 minutes of intermittent mixing, the extract was centrifuged, and supernatants containing nuclear extracts were secured. The protein content was measured by the Bradford method. If the nuclear extracts were not used immediately, they were stored at −80°C.

**Figure 4:**
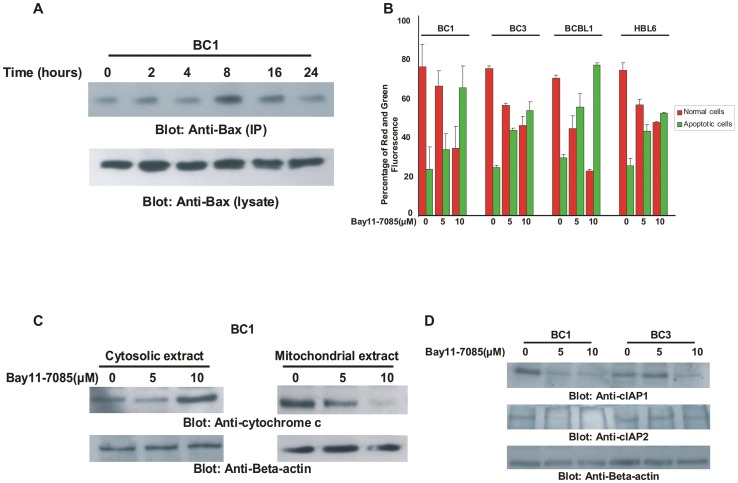
Bay11-7085 treatment of PEL cells activates mitochondrial apoptotic pathway in PEL cell lines. (**A**) **Bay11-7085-induced Bax activation in PEL cells.** After treating with 10 µM Bay11-7085 for indicated time periods, BC1 cells were lysed in 1% Chaps lysis buffer and subjected to immuno-precipitation with anti-Bax 6A7 antibody for detection of conformationally changed Bax protein. In addition, the total cell lysates were applied directly to SDS–PAGE, transferred to immobilon membrane and immuno-blotted with specific anti-Bax polyclonal antibody. (**B**) **Bay11-7085 treatment causes change in mitochondrial membrane potential in PEL cells.** PEL cells were treated with and without 5 and 10 µM Bay11-7085 for 24 hours. Live cells with intact mitochondrial membrane potential and dead cells with lost mitochondrial membrane potential was measured by JC-1 staining and analyzed by flow cytometry as described in Materials and Methods. (**C**) **Bay11-7085 treatment causes release of cytochrome c from mitochondria into cytosole in PEL cells.** BC1 cells were treated with 5 and 10 µM Bay11-7085 for 24 hours. Mitochondrial free cytosolic fractions and cytosolic extracts were isolated and immunoblotted with antibody against cytochrome c and Beta-actin. (**D**) **Bay11-7085 treatment causes down-regulation of IAPs in PEL cells.** BC1 and BC3 cells were treated with 5 and 10 µM Bay11-7085 for 24 hours. Cells were lysed and equal amounts of proteins were separated by SDS-PAGE, transferred to PVDF membrane, and immunoblotted with antibodies against cIAP1 and cIAP2. Beat actin was used for equal loading.

### Electrophoretic Mobility Shift Assay for NF-kB

The single-stranded 3′-end biotin-labeled probe containing the NFkBconsensus site 5′-AGTTGAGGGGACTTTCCCAGGC-3′, and 3′-TCAACTCCCCTGAAAG GGTCCG5′ were purchased from Metabion (Martinsried, Germany). The biotinylated oligonucleotides were annealed by denaturing at 90°C for 1 minute and cooled to room temperature for 1 hour. The EMSA binding reactions were performed by utilizing a LightShift chemiluminescent EMSA kit (Pierce, Rockford, IL) as described previously [27]. Specifically, 3 µg nuclear extract was incubated in 1Xbinding buffer containing 2.5% glycerol, 0.05% NP-40, 50 mM KCl, 5 mM MgCl2, 50 ng Poly (dI–dC) and biotinylated probe with or without protein extract for 30 min at room temperature. The complexes were separated on a 6% polyacrylamide–0.5XTris-borate-EDTA gel and transferred to a positive charge nylon membrane. After the transfer was completed, the membrane was cross-linked and biotin-labeled DNA was detected by using a chemiluminescent detection kit (Pierce, Rockford, IL).

### Measurement of Mitochondrial Potential and Cytochrome C Release

After treatment of PEL cell lines with two doses of Bay11-7085 for 48 hours, mitochondrial membrane potential was measured using JC1 dye and release of cytochrome c was analyzed using immunoblotting on cytosolic protein fractions as described previously [26].

**Figure 5:**
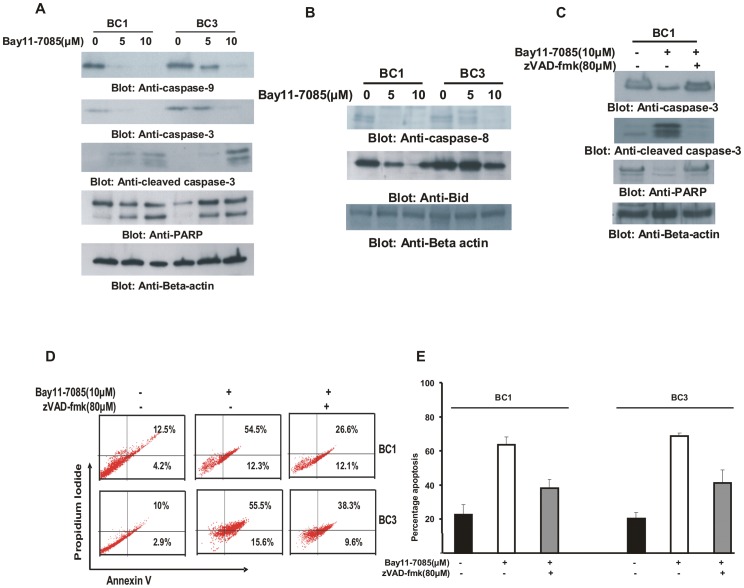
Bay11-7085 induced apoptosis is caspase dependent in PEL cell lines. (**A**) **Activation of caspases-9, -3, and cleavage of PARP induced by Bay11-7085 treatment in PEL cells.** BC1 and BC3 cells were treated with 5 and 10 µM Bay11-7085 for 24 hours. Cells were lysed and equal amounts of proteins were separated by SDS-PAGE, transferred to PVDF membrane, and immunoblotted with antibodies against caspase-9, caspase-3, cleaved caspase-3 and PARP. Beta-actin was used for equal loading. (**B**) **Bay11-7085 treatment causes cleavage of caspase-8 and truncation of Bid in PEL cells.** After treatment with 5 and 10 µM Bay11-7085 for 24 hours, cells were lysed, and equal amount of proteins were separated by SDS-PAGE, transferred to PVDF membrane, and immunoblotted with antibodies against caspase-8 and Bid. (**C, D and E**) **Bay11-7085-induced apoptosis is caspase dependent in PEL cells.** PEL cells were pre-treated with 80 µM zVAD-fmk for 2 hours and then treated with 10 µM Bay11-7085 for 24 hours. Following treatment, cells were either lysed and equal amounts of proteins were separated by SDS-PAGE, transferred to PVDF membrane, and immunoblotted with antibodies against caspase-3, cleaved caspase-3 and PARP (**C**) or stained with FITC conjugated annexin V/PI and analyzed by flow cytometry (**D**). Bar graph denotes percentage apoptosis from three independent experiments (**E**).

### Detection of Bax Conformational Changes

Cells were treated with 10 µM Bay11-7085 for various time periods and lysed with Chaps lysis buffer (10 mM HEPES (ph 7.4), 150 mM NaCl, 1% Chaps) and immuno-precipitated with anti-Bax-6A7 monoclonal antibody and Bax conformation was detected as described earlier [27].

### Gene Silencing Using siRNA

p65 siRNA was purchased from Santa Cruz Biotechnology, Inc. (Santa Cruz, CA, USA) while AKT siRNA and scrambled control siRNA were purchased from Qiagen (Valencia, CA, USA). Cells were transfected with above mentioned siRNA using Lipofectamine 2000 (Invitrogen, Carlsbad, CA) for 6 hours following which the lipid and siRNA complex was removed and fresh growth medium was added. Cells were either lysed 48 hours after transfection and specific protein levels were determined by Western Blot analysis with specific antibodies.

## Results

### NFkB is Constitutively Activated in PEL Cell Lines

The NFkB survival pathway has been found to be constitutively activated in various cancer cells [29], [30], [31], but its role in PEL cells is unknown. We examined the activation of status of NFkB in different PEL cell lines. Constitutive activation of NFkB is determined by the presence of p65 sub unit of NFkB in the nuclear compartment of cells where it exerts its transcriptional activity [32]. We found that all four PEL cell lines studied exhibited constitutive activation of NFkB detected by EMSA assays (Figure 1A). We next sought to determine whether treatment of PEL cells with a specific inhibitor of NFκB; Bay11-7085 could inhibit translocation of p65 into nucleus. BC1 and BC3 cell lines were treated at 5 or 10 µM final concentrations of Bay11-7085 for 24 hours and nuclear extracts were prepared and EMSA was performed. As shown in Figure 1B, Bay11-7085 treatment caused decreased expression of p65 in the nuclear compartment suggesting that activation of NFkB pathway can be blocked in PEL cells following treatment with Bay11-7085.

**Table 1:** Combination index calculation using Chou and Talalay method in PEL cell lines.

BC1					
Bay11-7085(µM)	LY294002(µM)	Combinationndex (CI)	Fractionaleffect (Fa)	Dose ReductionBay11-7085 (µM)	Index (DRI)LY294002 (µM)
0.5	1.0	0.391	0.254	5.96	4.48
1.0	5.0	0.125	0.74	11.7	25.09
5.0	10	0.387	0.732	5.60	4.78
10	25	0.664	0.776	2.89	3.13
25	50	1.295	0.797	1.65	1.44
**BC3**					
**Bay11-7085** **(µM)**	**LY294002** **(µM)**	**Combination** **ndex (CI)**	**Fractional** **effect (Fa)**	**Dose Reduction** **Bay11-7085 (µM)**	**Index (DRI)** **LY294002 (µM)**
0.5	1.0	0.701	0.17	2.89	2.81
1.0	5.0	0.177	0.693	8.24	17.87
5.0	10	0.582	0.671	3.68	3.21
10	25	1.327	0.667	1.44	1.57
25	50	1.876	0.754	1.15	0.98

### Bay 11-7085 Inhibits IκBα Phosphorylation in PEL Cells

The degradation of IκBα and subsequently release of NFkB (p65) requires prior phosphorylation at Ser32 and Ser36 residues [33]. Bay11-7085 has been shown to selectively and irreversibly inhibit the phosphorylation of IκBα [27]. We therefore investigated whether Bay11-7085 treatment of PEL cell lines altered phosphorylation of IκBα. BC1 and BC3 cells were treated with 5 and 10 µM Bay11-7085 for 24 hours and proteins were separated on SDS-PAGE and immunoblotted with antibodies against p-IκBα and actin. As shown in Figure 1C, in untreated samples, there was constitutive expression of Ser32-phosphorylated IκBα, while treatment of Bay11-7085 dephosphorylated IκBα in a dose-dependent manner in both cell lines.

**Figure 6:**
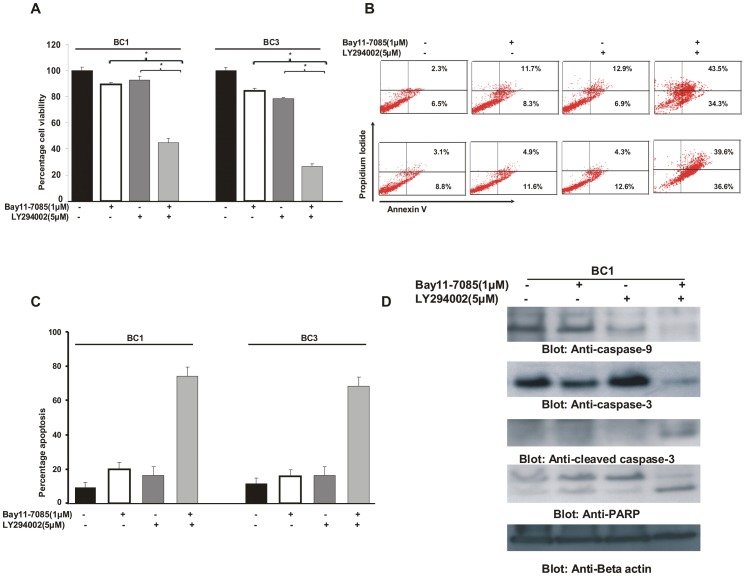
Combination of NFκB and PI3-kinase/AKT inhibitors induce synergistic inhibition of cell viability and induction of apoptosis in PEL cell lines. (**A**) **Combination of sub-toxic doses of Bay11-7085 and LY294002 suppresses growth of PEL cells.** BC1 and BC3 cells were treated with either 1 µM Bay11-7085 or 5 µM LY294002 alone or in combination for 24 hours. Cell viability was measured by MTT assays as described in Materials and Methods. The graph displays the mean +/− SD (standard deviation) of three independent experiments, * p<0.05, statistically significant (Students *t*-test). (**B and C**) **Combination of Bay11-7085 and LY294002 induces efficient apoptosis in PEL cells.** BC1 and BC3 cells were treated with either 1 µM Bay11-7085 or 5 µM LY294002 alone or in combination for 24 hours. Following treatment, cells were stained with FITC conjugated annexin V/PI and cells were analyzed by flow cytometry as described in material and methods (**B**). Bar graph denotes percentage apoptosis of three independent experiments (**C**). (**D**) **Combination of sub-toxic doses of Bay11-7085 and LY294002 induced apoptosis is caspase dependent.** BC1 cells were treated with either 1 µM Bay11-7085 or 5 µM LY294002 alone or in combination for 24 hours. Following treatment, cells were lysed and equal amounts of proteins were separated by SDS-PAGE, transferred to PVDF membrane, and immunoblotted with antibodies against caspase-9, caspase-3, cleaved caspase-3 and PARP. Beta-actin was used for equal loading.

### Bay 11-7085 Down-regulates Expression of NFkB-regulated Proteins in PEL Cells

Previous studies have shown that NFkB activation regulates a number of survival genes including IκBα, Bcl-2, Bcl-Xl, XIAP and Survivin [34]. Therefore, we studied the effect of Bay11-7085 on the expression of these proteins by immunoblotting with specific antibodies. Following treatment with Bay11-7085 for 24 hours, proteins were isolated from BC1 and BC3 cells and processed for immunoblotting. As shown in Figure 1D, Bay11-7085 down-regulated the expression of IκBα, Bcl-2, Bcl-Xl and XIAP in a dose dependent manner in both cell lines. To confirm for specificity of the findings using Bay11-7085, we transfected either p65 specific siRNA or scrambled non-specific siRNA in BC1 and BC3 cells. After 48 hours of transfection, proteins were extracted from the cells and immunoblotted with antibodies against IκBα, Bcl-2, Bcl-xL and XIAP. There was concordance in the data generated following treatment with Bay11-7085 and siRNA against p65. As shown in Figure 1E, siRNA knockdown of p65 led to down-regulation of IκBα, Bcl-2, Bcl-xL and XIAP in both cell lines. These data clearly suggest that inhibition of NFkB leads to suppression of expression of genes that are involved in growth and survival of PEL cells.

### Inhibition of NFkB Inactivates AKT and its Down-stream Targets in PEL Cells

It has been previously shown that NFkB survival pathway is also linked to other survival pathways including PI3-kinase/AKT pathway in various cancers [18], [19]. However, cross-talk between these two pathways has not been elucidated in PEL cells. Therefore, we determined whether there are any interactions between NFkB and the PI3-kinase/AKT pathway. For this reason, BC1 and BC3 cells were treated with Bay11-7085 and the extracted proteins were immunoblotted with antibodies against p-AKT, p-Foxo1, p-GSK3 and p-Bad. As shown in Figure 2A, Bay11-7085 treatment of PEL cells inactivated AKT and caused de-phosphorylation of its downstream targets Foxo1, GSK3 and Bad. To confirm the Bay11-7085 findings, we examined the effects of p65 knockdown in PEL cells. For that purpose, we treated PEL cells with siRNA targeting p65. siRNA knockdown of p65 led to inactivation of AKT and its downstream targets, FOXO1, GSK3 and Bad (Figure 2B). In addition, we found that in PEL cells in which AKT expression had been knocked down by specific siRNA targeted against AKT, there was inactivation of IκBα suggesting a cross-talk between the NFκB and PI3-kinase/AKT pathway (Figure 2C).

### Bay11-7085 Inhibits Cell Viability and Induces Apoptosis of PEL Cells

Since we have shown that Bay11-7085 treatment of PEL cells causes down-regulation of NFκB and PI3-kinase/AKT pathway, we sought to determine whether these effects cause inhibition of cell viability and induce apoptosis in PEL cells. BC1, BC3, BCBL, and HBL6 cells were cultured in the presence or absence of 0, 1, 10 and 25 µM Bay11-7085 for 24 hours and cell viability was subsequently assessed. As shown in Figure 3A, Bay11-7085 treatment caused inhibition of cell viability in a dose dependent manner in all the PEL cell lines. We next sought to determine whether inhibition of cell viability occurred due to PEL cells undergoing cell death. For this reason, we treated PEL cell lines with Bay11-7085 for 24 hours and after staining them with calcein and ethidium homodimer, plasma membrane integrity was assessed. As shown in Figure 3B, untreated cells were stained green depicting alive cells with plasma membrane integrity intact while cells treated with Bay11-7085 showed an increase in red cells suggesting disruption of plasma membrane integrity, i.e., dead cells. We further analyzed PEL cells for cell cycle fractions after treatment with Bay11-7085 for 24 hours and found that Bay11-7085 increased cells in subG1/Apo fraction in all the cell lines tested (Figure 3C). This data suggested that Bay11-7085 was inducing apoptosis in PEL cell lines. To further confirm that this increase in the sub-G1 population indeed reflected apoptosis, PEL cells were treated with 5 and 10 µM Bay11-7085 as indicated and apoptotic cells were analyzed by annexin V dual staining. As shown in Figure 3D, Bay11-7085 treatment resulted in apoptosis in a dose dependent manner in all PEL cell lines. We also confirmed apoptosis by DNA fragmentation in BC1 and BC3 cells treated with Bay11-7085 for 24hours (data not shown).

Finally, we also treated five normal peripheral blood mononuclear cells (PBMNC) from healthy donors with 5 and 10 µM Bay11-7085 to assess the effect of Bay11-7085 on normal cells. As shown in Figure S1, there was minimal apoptotic response (2–16%) in all the PBMNC samples following treatment with 5 and 10 µM Bay11-7085. These data suggest that Bay11-7085 induces apoptosis in PEL cells and is non-toxic to normal PBMNCs.

### Bay11-7085 Activates the Intrinsic Apoptotic Pathway in PEL Cells

Bay11-7085 treatment of PEL cells has the ability to down-regulate the expression of anti-apoptotic and pro-survival targets of p65 that play major roles in the prevention of apoptosis and survival of cancer cells; it also has the ability to inactivate the expression of Bad, a down-stream target of AKT [35]. Once Bad is inactivated, native Bad exerts its pro-apoptotic action to activate the intrinsic apoptotic pathway by initially inducing conformational changes in Bax protein. To confirm this role of Bad, BC1 cells were treated withBay11-7085 for various time periods and the cells were lysed in 1.0% Chaps lysis buffer and lysates were immunoprecipitated with an anti-BAX 6A7 antibody that recognizes only the conformationally changed Bax protein. The detergent Chaps has been shown to retain the Bax protein in its native conformation [36]. As shown in Figure 4A, conformationally changed Bax was increased after 8 hours of treatment with Bay11-7085, while the signal decreased after 24 hours treatment. We next determined whether Bax conformational changes lead to changes in the mitochondrial membrane potential in PEL cells. All four PEL cell lines were treated with Bay11-7085 for 24 hours and cells were stained with JC1, a surrogate marker for detection of changes in mitochondrial membrane potential, followed by flow cytometry analysis. If the mitochondria are intact, JC1 dye enters them, remains in the monomeric form and stains the cell red, however, if the mitochondria are damaged, JC1 cannot be retained in the mitochondria and is expelled in the cytoplasm in the polymeric form and stains the cell green. As shown in Figure 4B, Bay11-7085 treatment resulted in loss of mitochondrial membrane potential in all the PEL cell lines as measured by increase in JC1 stained green florescence depicting apoptotic cells. We then studied the release of cytochrome c from mitochondria into cytosol. As shown in Figure 4C, higher levels of cytochrome c were detactable in cytosolic and lower levels in the mitochondrial fraction in the BC1 cell line after Bay11-7085 treatment. These findings suggest that Bay11-7085 treatment of PEL cell lines causes dephosphorylation of Bad leading to activation of the mitochondrial apoptotic pathway.

### Down-regulation of Inhibitor of Apoptosis Proteins (IAPs) following Treatment of PEL Cells with Bay11-7085

IAPs play an important role in inhibition of apoptosis by disrupting the recruitment of caspases-9 and -3 for efficient apoptosis to occur. We found that in addition to down-regulation of XIAP and Survivin, Bay11-7085 treatment also inhibited other IAPs including cIAP1 and cIAP2 (Figure 4D).

### Bay11-7085 Treatment Induces Activation of Caspases in PEL Cells

Since caspases are important mediators of apoptosis in response to various apoptotic stimuli [13], [26], [37], we investigated whether Bay11-7085 treatment also causes their activation and cleavage in PEL cells. BC1 and BC3 cells were treated with Bay11-7085 for 24 hours and cell lysates were immunoblotted with antibodies against caspase-9, caspase-3, cleaved caspase-3 and PARP. As shown in Figure 5A, Bay11-7085 treatment of PEL cells induced activation of caspase-9 and caspase-3 cleavage in both cell lines. PARP-1, a downstream target of caspase-3 was also cleaved in bothcell lines, a hallmark of cells undergoing apoptosis. It has been shown by us and others that caspase-3 has the ability to activate caspase-8 and cleave Bid, downstream of the mitochondrial pathway to potentate the apoptotic signal [38]. Therefore, we sought to determine the expression of caspase-8 and Bid following treatment with Bay11-7085. As shown in Figure 5B, Bay11-7085 treatment resulted in reduction in the intensity of the full-length band of pro-caspase-8 indicating activation of caspase-8 in both cell lines. Bid is a BH3-proapoptotic protein that can be cleaved directly by caspases-8 during apoptosis. The cleaved or truncated Bid also plays a role in the induction of Bax conformational change and subsequent translocation to mitochondria [39], [40]. Therefore, we examined if Bay11-7085 treatment of PEL cells could truncate the Bid protein. As shown in Figure 5B, there was decreased expression of Bid protein following treatment of BC1 and BC3 cell lines with Bay11-7085 suggesting that caspase-8 and Bid are also activated following inhibition of NFκB survival pathway. Furthermore, pre-treatment of PEL cells with 80 µM z-VAD-fmk a universal inhibitor of caspases markedly decreased caspase-3 activation (Figure 5C), and prevented apoptosis (Figure 5D and E) induced by Bay11-7085, clearly indicating that caspases play a critical role in Bay11-7085-induced apoptosis in PEL cells. Finally, to confirm whether the intrinsic apoptotic pathway is actually activated in Bay11-7085 induced apoptosis, we pre-treated BC1 cells with either 80 µM caspase-8 inhibitor (IETD-CHO) or 80 µM caspase-9 inhibitor (LEHD-CHO) for 3 hours followed by treatment with 10 µM Bay11-7085 for 24 hours. Following treatment, cells were lysed and proteins were extracted, immunoblotted and probed with different antibodies. As shown in Figure S2A, caspase-8 inhibitor blocked cleavage of caspase-8 and Bid but failed to block cleavage of caspase-3. Alternately, caspase-9 inhibitor blocked cleavage of caspase-9, caspase-3, caspase-8 and Bid in BC1 cells (Figure S2B). These set of data clearly indicates that Bay11-7085 treatment induces apoptosis via intrinsic apoptotic pathway.

### Combination Treatment of PEL Cells with PI3-kinase/AKT Inhibitor and Bay11-7085 Induce Synergistically Potent Apoptosis

Cross-talk between NFkB pathway and PI3-kinase/AKT pathway has been shown in various cancers [18], [19], however, the relationship between these two pathways have not been fully explored in PEL cells. We found that inhibition of NFkB pathway by Bay11-7085 not only inactivates the NFkB pathway, it also inactivates AKT as well as down-stream targets of AKT such as FOXO1, GSK3 and Bad. Utilizing this information, we aimed at targeting PEL cell lines with a combination of NFkB and PI3-kinase/AKT inhibitors at sub-optimal doses to determine the synergistic therapeutic potential of such a combination. Multiple experiments were conducted to define optimal doses for a synergistic apoptotic response of the combination of Bay11-7085 and LY294002, a specific PI3-kinase inhibitor. As shown in Table 1 and Figure S3, using Chou and Talalay method [41], we found that 1 µM Bay11-7085 and 5 µM LY294002 exerted the maximum synergistic apoptotic response in BC1 cells (combination index 0.125) and BC3 cells (combination index 0.177), with both the values being less than 1.0 suggesting a strong synergistic response [41]. Using these doses, we first assessed the cell viability of cells following treatment with combination of Bay11-7085 with LY294002. As shown in Figure 6A, neither Bay11-7085 at a concentration of 1 µM nor LY294002 at a concentration of 5 µM could inhibit cell viability in any of the PEL cell lines. However when both the drugs were added together to the cultures as a combination, there was efficient inhibition of cell viability in PEL cells. Next, we examined the apoptotic response of PEL cells following combination treatment with Bay11-7085 and LY294002. As shown in Figure 6B and C, Bay11-7085 or LY294002 alone failed to induce apoptosis at sub-optimal doses, however, when both drugs were used in combination there was a synergistic apoptotic response as detected by annexin V/PI dual staining. Finally, we found that combination of Bay11-7085 and LY294002 induced apoptosis via the activation of the caspase cascade and cleavage of PARP (Figure 6D).

## Discussion

Primary effusion lymphoma (PEL) is a very aggressive and fatal human malignancy, which frequently and rapidly develops resistance to conventional chemotherapeutic agents [3]. It is now postulated that the mechanisms of lymphomagenesis involve deregulation of several signaling pathways that may interact with each other to escape programmed cell death [18], [19]. We have previously shown that the PI3-kinase/AKT pathway is activated in PEL cells and is one of the driving forces behind the aggressive phenotype of these cells [12]. In addition to the PI3-kinase/AKT pathway, PEL cells are also dependent on other activated signaling pathways including NFκB [42], [43]. In this study, we have investigated the molecular mechanism of NFκB mediated anti-apoptotic role in PEL cell lines using Bay11-7085, a specific inhibitor of NFκB pathway as well as siRNA knockdown targeting the p65 subunit. Our data suggest that inhibition of NFκB causes inability of the p65 subunit to enter the nucleus resulting in down-regulation of anti-apoptotic targets of p65 such as IκBα, XIAP, Bcl-Xl and Survivin. In addition, Bay11-7085 treatment also led to inactivation of PI3-kinase/AKT pathway. Genetic knockdown of p65 with siRNA also resulted in inactivation of PI3-kinase/AKT signaling suggesting a cross-talk between these two survival pathways in PEL cells. Cross-talk between these two pathways has been shown in other cancers. Reciprocally, siRNA knockdown of AKT also led to dephosphorylation of IκBα confirming the presence of a cross-talk between the survival pathways.

Apoptosis is a multi step process and an increasing number of genes have been identified that are involved in the control or execution of apoptosis [44]. Inhibition of apoptosis is a key mechanism used by cancer cells to proliferate and grow unabated. Activation of NFκB pathway also plays a major role in inhibiting apoptosis by causing up-regulation of key anti-apoptotic proteins such as Bcl-2, Bcl-Xl, XIAP and Survivin [14]. In order for efficient apoptosis to occur, these anti-apoptotic proteins need to be down-regulated so that the apoptotic pathway is activated and the cells die. Our studies established that Bay-11-7085 treatment down-regulates the expression of Bcl-2 and Bcl-Xl protein and inactivated Bad protein that in turn allowed Bax conformational changes and subsequent translocation to mitochondria [45] leading to the formation of mitochondrial pores. These events result in loss of mitochondrial potential and release of cytochrome c from mitochondria to cytoplasm [46]. Cytochrome c release from the mitochondria has been proposed as the most critical event for cells to initiate the apoptotic cascade [47].

Our data also established that Bay11-7085 treatment causes down-regulation of IAPS; XIAP and Survivin that interfere with the activation and cleavage of caspases [48]. Once these IAPs were down-regulated along with release of cytochrome c following inhibition of NFkB activity by Bay11-7085, there was sequential activation of caspase-9, caspase-3 and cleavage PARP in PEL cell lines. Furthermore, pre-treatment of PEL cells with a broad-spectrum caspase inhibitor markedly blocked Bay11-7085-induced apoptosis. These data clearly suggests that activation of caspase-cascades is more prominently involved in Bay11-7085-induced apoptosis.

In this manuscript, we have hypothesized that a functional link might exist between AKT and NFkB pathways in the pathogenesis of PEL and activation of these survival pathways may sustain survival and proliferation of these malignant cells. Since our findings suggested that NFkB and AKT pathways are activated in PEL cells, we proposed that simultaneous targeting of these pathways may synergistically induce apoptosis of PEL cells. We found that when PEL cell lines treated with sub-toxic doses of Bay11-7085 and LY294002, a specific inhibitor of PI3-kinase/AKT, neither Bay11-7085 at a dose of 1 µM nor LY294002 at 5 µM could inhibit cell viability in any of the PEL cell lines. However when both the drugs were given together as a combination, there was efficient inhibition of cell viability of PEL cells via induction of caspase-mediated apoptosis. These data clearly indicate the importance of targeting multiple survival pathways simultaneously using sub-toxic doses of specific inhibitors thereby decreasing the chances of toxicity and increasing the response to therapy.

Despite advances in therapeutic regimes for the treatment of aggressive NHL over the last decade, PELs are still refractory to conventional systemic chemotherapy with a mean overall survival of 3 months [49]. The suggested benefit of high-dose Methotrexate in association with CHOP (Cyclophosphamide, Doxorubicin, Prednisolone and Vincristine)-like regimens is negatively balanced by the hampered toxicity of Methotrexate in the presence of serous effusions [3]. Therefore, newer therapeutic agents such as Bay11-7085 and LY294002 may play important roles in the management of these aggressive lymphomas in combination with conventional chemotherapy to improve survival and decrease toxicity.

In conclusion, our results demonstrate that down-regulation of the NFκB pathway leads to activation of the mitochondrial apoptotic pathway. This process occurs via down-regulation of downstream targets of p65; Bcl-2, Bcl-Xl, XIAP and Survivin, ultimately resulting in activation of caspase-dependent apoptosis. Simultaneously, we also investigated the cross-talk between NFκB and PI3-kinase/AKT pathway and found that targeting of these survival pathways simultaneously significantly increases the apoptotic stimuli in PEL cells thereby decreasing the chances of toxicity. Taken altogether our data suggests that Bay11-7085 possesses the chemopreventive/therapeutic potentials against these aggressive lymphomas either alone or in combination with other inhibitors.

## Supporting Information

Figure S1 **Bay11-7085 treatment is non-toxic to normal peripheral blood mononuclear cells (PBMNC).** (**A**) PBMNC from 5 normal healthy donors were isolated and treated with 5 and 10 µM Bay11-7085 for 24 hours. Following treatment, cells were harvested and stained with fluorescein conjugated annexin V/PI and cells were analyzed by flow cytometry. Bar graph denotes an average of 3 independent experiments.
(TIF)

Figure S2 **Bay11-7085-induced apoptosis is via intrinsic apoptotic pathway in PEL cells.** BC1 cells were pre-treated with either 80 µM IETD-CHO (caspase-8 inhibitor) **(A)** or 80 µM LEHD-CHO (caspase-9 inhibitor) **(B)** for 3 hours followed by treatment with 10 µM Bay11-7085 for 24 hours. Following treatment, proteins were extracted, immunoblotted and probed with antibodies against caspase-8, caspase-9, caspase-3 and Bid. Beta-actin was used to insure equal loading.
(TIF)

Figure S3 **Synergistic apoptotic response of Bay11-7085 and TRAIL in PEL cells.** BC1 and BC3 cells were treated with various combinations of Bay11-7085 and TRAIL for 24 hours and dose effect (**A and C**) and Fractional effect (**B and D**) graphs were generated using Calcusyn software. Apoptotic response analysis was measured as mean ± SD values normalized to control. Combination indices were calculated using Chou and Talalay methodology.
(TIF)
