# Correction: Cross-Talk between NFkB and the PI3-Kinase/AKT Pathway Can Be Targeted in Primary Effusion Lymphoma (PEL) Cell Lines for Efficient Apoptosis

**DOI:** 10.1371/journal.pone.0092484

**Published:** 2014-03-10

**Authors:** The PLOS ONE Staff

After the publication of this manuscript we observed inaccuracies in several of the figures. The concerns noted are as below:

Figure 1C in the article displays the same beta-actin control lanes as Figure 2E in the article "Phosphorylated IκBα Predicts Poor Prognosis in Activated B-Cell Lymphoma and Its Inhibition with Thymoquinone Induces Apoptosis via ROS Release" (10.1371/journal.pone.0060540). The actin control in the article 10.1371/journal.pone.0060540 is correct; Figure 1C in the article 10.1371/journal.pone.0039945 is duplicated in error.

**Figure 1:**
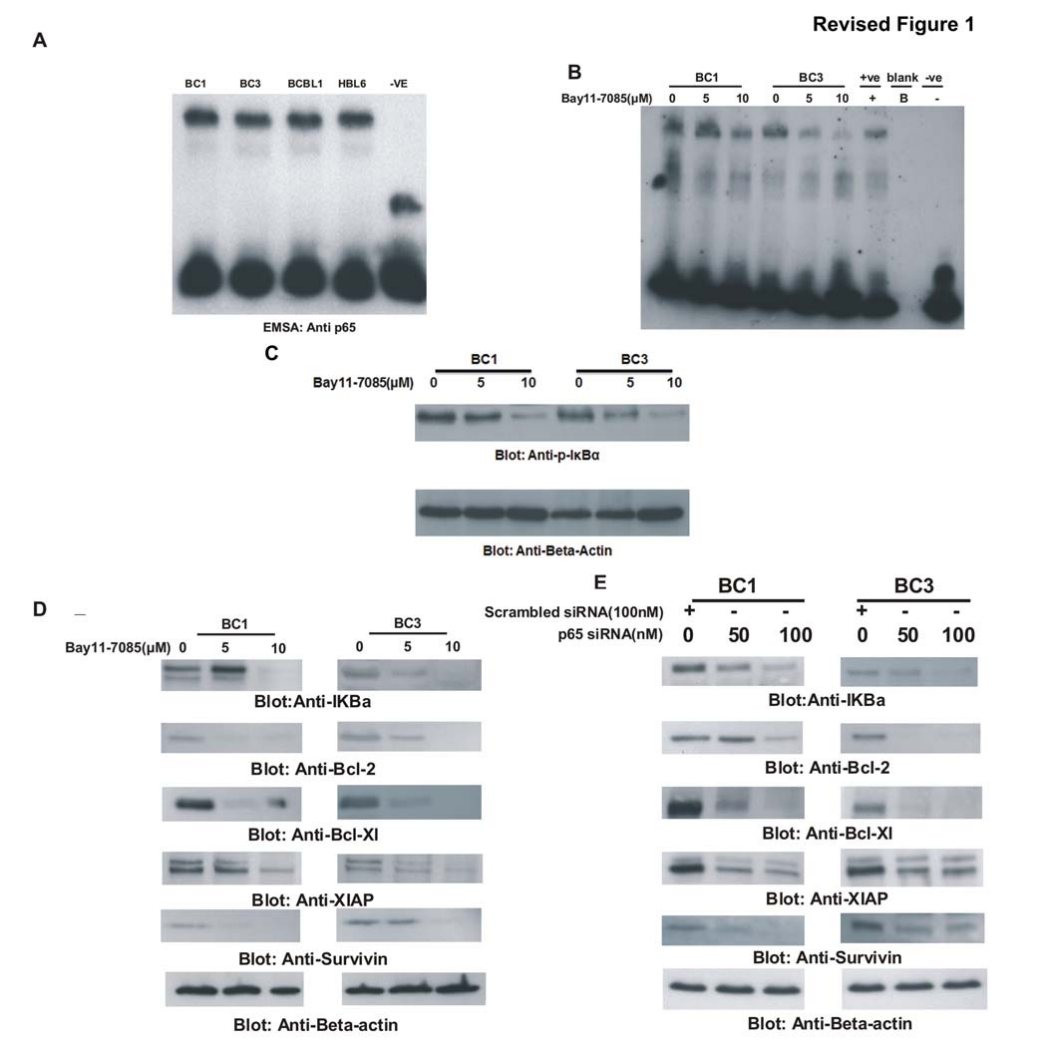
Role of NFkB in PEL cell lines (A) Constitutive expression of NFkB in PEL cells. Nuclear extracts from BC1, BC3, BCBL1 and HBL6 cell lines were prepared as described in material and Methods and electrophoretic mobility shift assay (EMSA) was performed as described in Materials and Methods. Briefly, 5x10^6^ cells were washed with cold PBS and suspended in 0.4 mL hypotonic lysis buffer containing protease inhibitors for 30 minutes. The cells were then lysed with 10% Nonidet P-40. **(B)** **Bay11-7085 inhibits constitutive nuclear NFkB in PEL cells.** BC1 and BC3 cells were treated with 5 and 10 μM Bay11-7085 for 24 hours. Nuclear extracts were prepared and EMSA was performed. **(C) Effect of Bay11-7085 on IkBa phosphorylation in PEL cells.** BC1 cells were treated with 5 and 10 μM Bay11-7085 for 24 hours. Cells were lysed and equal amounts of proteins were separated by SDS-PAGE, transferred to PVDF membrane, and immunoblotted with antibodies against phospho-IkBa and Beta actin as indicated. **(D) Bay11-7085 treatment causes down-regulation of expression of down-stream targets of p65.** BC1 and BC3 cells were treated with 5 and 10 μM Bay11-7085 for 24 hours. Cells were lysed and equal amounts of proteins were separated by SDS-PAGE, transferred to PVDF membrane, and immunoblotted with antibodies against IkBa, Bcl-2, Bcl-Xl, XIAP, Survivin and Beta-actin. **(E) Transcriptional down-regulation of p65 causes decreased expression of p65 targets in PEL cells.** BC1 and BC3 cells were transfected with siRNA against p65 for 48 hours. Following transfection, cells were lysed and equal amounts of proteins were separated by SDS-PAGE, transferred to PVDF membrane, andimmunoblotted with antibodies against IkBa, Bcl-2, Bcl-Xl, XIAP, Survivin and Beta-actin.

**Figure 2:**
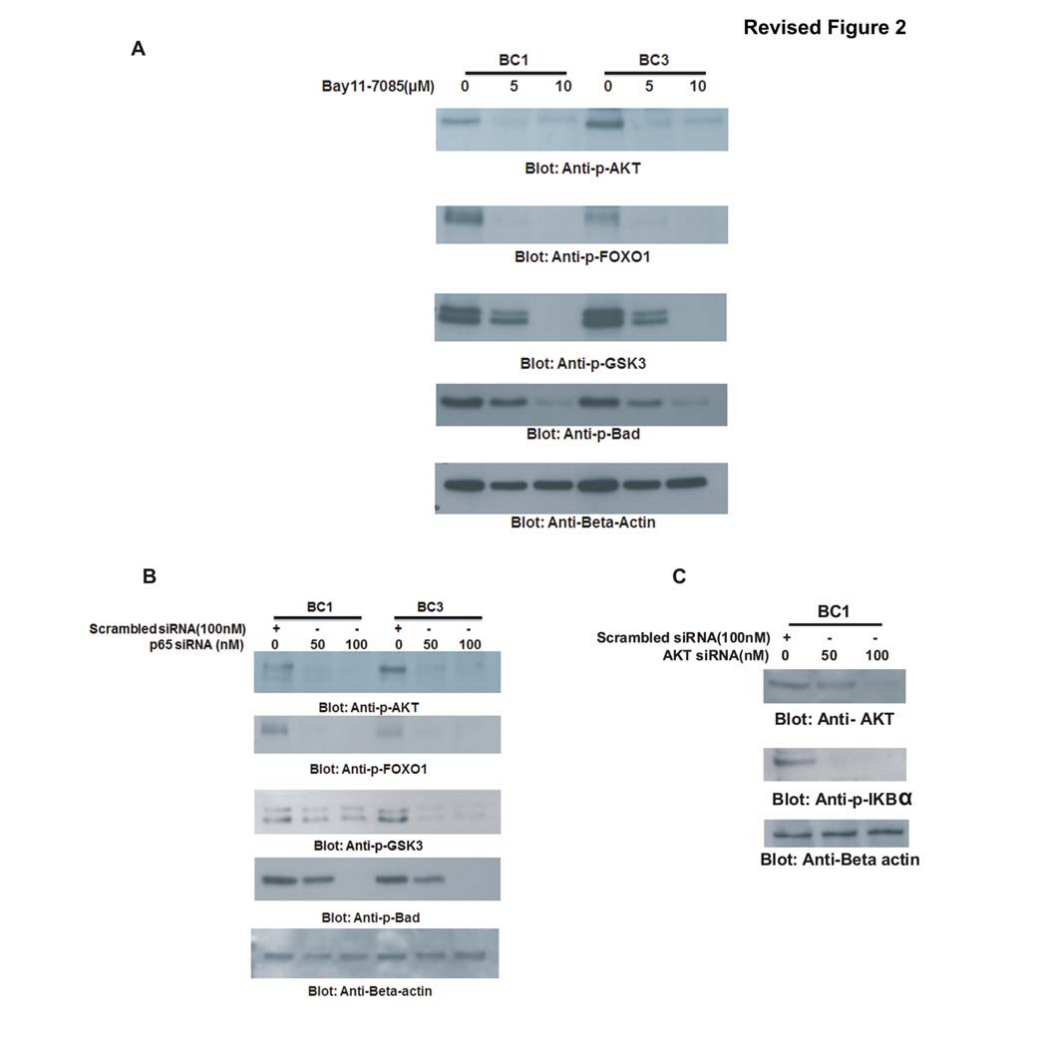
Cross-talk between NFkB and PI3-kinase/AKT pathway in PEL cell lines. (A) Bay11-7085 treatment inactivates AKT and its down-stream targets in PEL cells. BC1 and BC3 cells were treated with 5 and 10 μM Bay11-7085 for 24 hours. Cells were lysed and equal amounts of proteins were separated by SDS-PAGE, transferred to PVDF membrane, and immunoblotted with antibodies against p-AKT, p-FOXO1, p-GSK3, p-Bad and Beta-actin. **(B)** **Transcriptional knock down of p65 causes in-activation of AKT and its down-stream targets in PEL cells.** BC1 and BC3 cells were transfected with siRNA against p65 for 48 hours. Following transfection, cells were lysed and equal amounts of proteins were separated by SDS-PAGE, transferred to PVDF membrane, and immunoblotted with antibodies against p-AKT, p-FOXO1, p-GSK3, p-Bad and Beta-actin. **(C) Transcriptional targeting of AKT causes in-activation of NFkB pathway.** BC1 cells were transfected with siRNA against AKT for 48 hours. Following transfection, cells were lysed and equal amounts of proteins were separated by SDS-PAGE, transferred to PVDF membrane, and immunoblotted with antibodies against p-AKT, p-IkBa and Beta-actin.

The right column in Figure 2B displays the same beta-actin control as Figure 5C.

**Figure 5:**
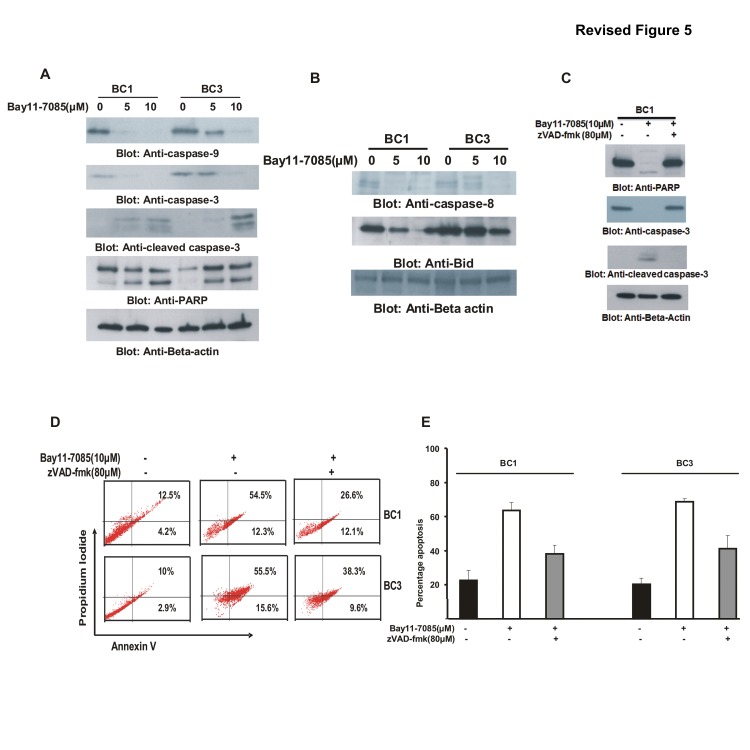
Bay11-7085 induced apoptosis is caspase dependent in PEL cell lines. (A) Activation of caspases-9, -3, and cleavage of PARP induced by Bay11-7085 treatment in PEL cells. BC1 and BC3 cells were treated with 5 and 10 μM Bay11-7085 for 24 hours. Cells were lysed and equal amounts of proteins were separated by SDS-PAGE, transferred to PVDF membrane, and immunoblotted with antibodies against caspase-9, caspase-3, cleaved caspase-3 and PARP. Beta-actin was used for equal loading. **(B) Bay11-7085 treatment causes cleavage ofcaspase-8 and truncation of Bid in PEL cells.** After treatment with 5 and 10 μM Bay11-7085 for 24 hours, cells were lysed and equal amount of proteins were separated by SDS-PAGE, transferred to PVDF membrane, and immunoblotted with antibodies against caspase-8 and Bid. **(C, D and E) Bay11-7085-induced apoptosis is caspase dependent in PEL cells.** PEL cells were pre-treated with 80 μM zVAD-fmk for 2 hours and then treated with 10 μM Bay11-7085 for 24 hours. Following treatment, cells were either lysed and equal amounts of proteins were separated by SDSPAGE, transferred to PVDF membrane, and immunoblotted with antibodies against caspase-3, cleaved caspase-3 and PARP **(C)** or stained with FITC conjugated annexin V/PI and analyzed by flow cytometry **(D).** Bar graph denotes percentage apoptosis from three independent experiments **(E).**

The concerns were raised to the attention of King Faisal Specialist Hospital and Research Center which investigated the concerns and established that the first author had made use of the incorrect actin panels in this publication.

The authors have repeated the experiments for the affected figures and have supplied corrected figures including the novel data for Figures 1C, 2E and 5C. The raw blots for these figures are also provided as supporting information as part of this correction.

The data in the corrected figures supports the results and conclusions as originally reported in the article. The authors apologize for the inaccurate representation of the data in the original figures.

## Supporting Information

File S1 Raw blots for Figure 1C
(ZIP)

File S2 Raw blots for Figure 2A
(ZIP)

File S3 Raw blots for Figure 2B
(ZIP)

File S4 Raw blots for Figure 5C
(ZIP)
