# Supplementary figures and images for: Cross-Talk between NFkB and the PI3-Kinase/AKT Pathway Can Be Targeted in Primary Effusion Lymphoma (PEL) Cell Lines for Efficient Apoptosis
Source: PLoS One. 2012 Jun 29;7(6):e39945. doi: 10.1371/journal.pone.0039945 (PMC3386924; doi:10.1371/journal.pone.0039945)

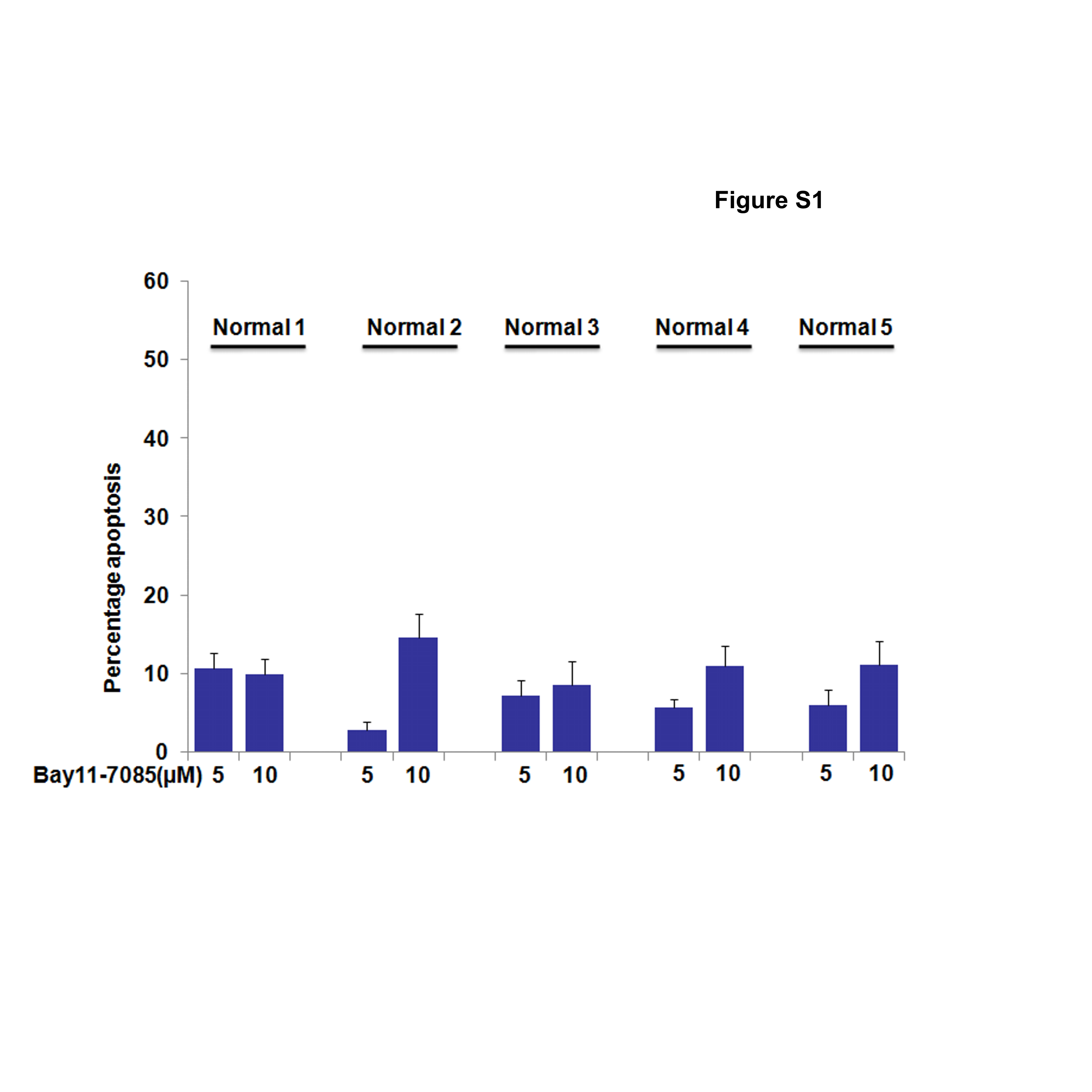

Supplement: Figure S1 — Bay11-7085 treatment is non-toxic to normal peripheral blood mononuclear cells (PBMNC). (A) PBMNC from 5 normal healthy donors were isolated and treated with 5 and 10 µM Bay11-7085 for 24 hours. Following treatment, cells were harvested and stained with fluorescein conjugated annexin V/PI and cells were analyzed by flow cytometry. Bar graph denotes an average of 3 independent experiments. (TIF) [file pone.0039945.s001.tif]

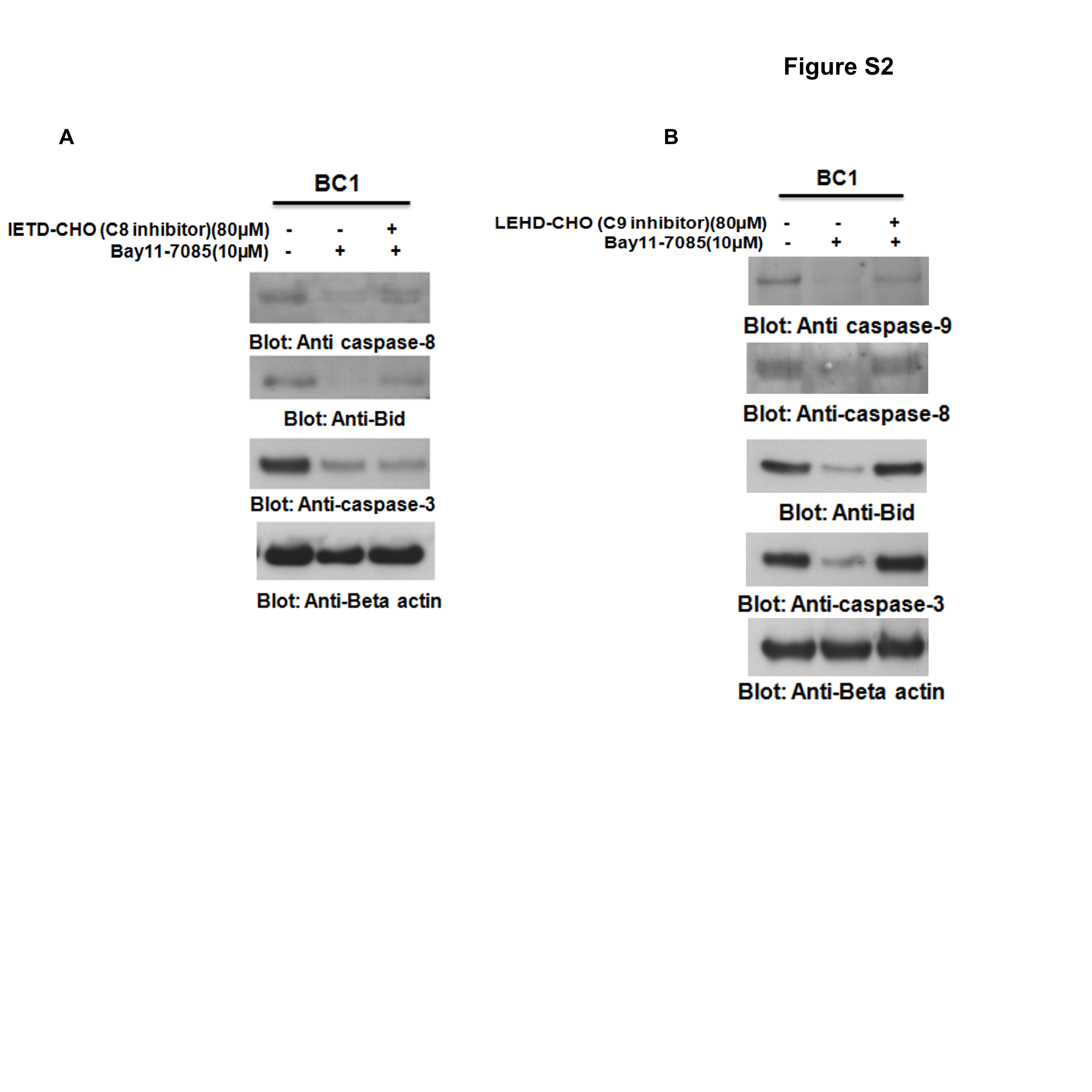

Supplement: Figure S2 — Bay11-7085-induced apoptosis is via intrinsic apoptotic pathway in PEL cells. BC1 cells were pre-treated with either 80 µM IETD-CHO (caspase-8 inhibitor) (A) or 80 µM LEHD-CHO (caspase-9 inhibitor) (B) for 3 hours followed by treatment with 10 µM Bay11-7085 for 24 hours. Following treatment, proteins were extracted, immunoblotted and probed with antibodies against caspase-8, caspase-9, caspase-3 and Bid. Beta-actin was used to insure equal loading. (TIF) [file pone.0039945.s002.tif]

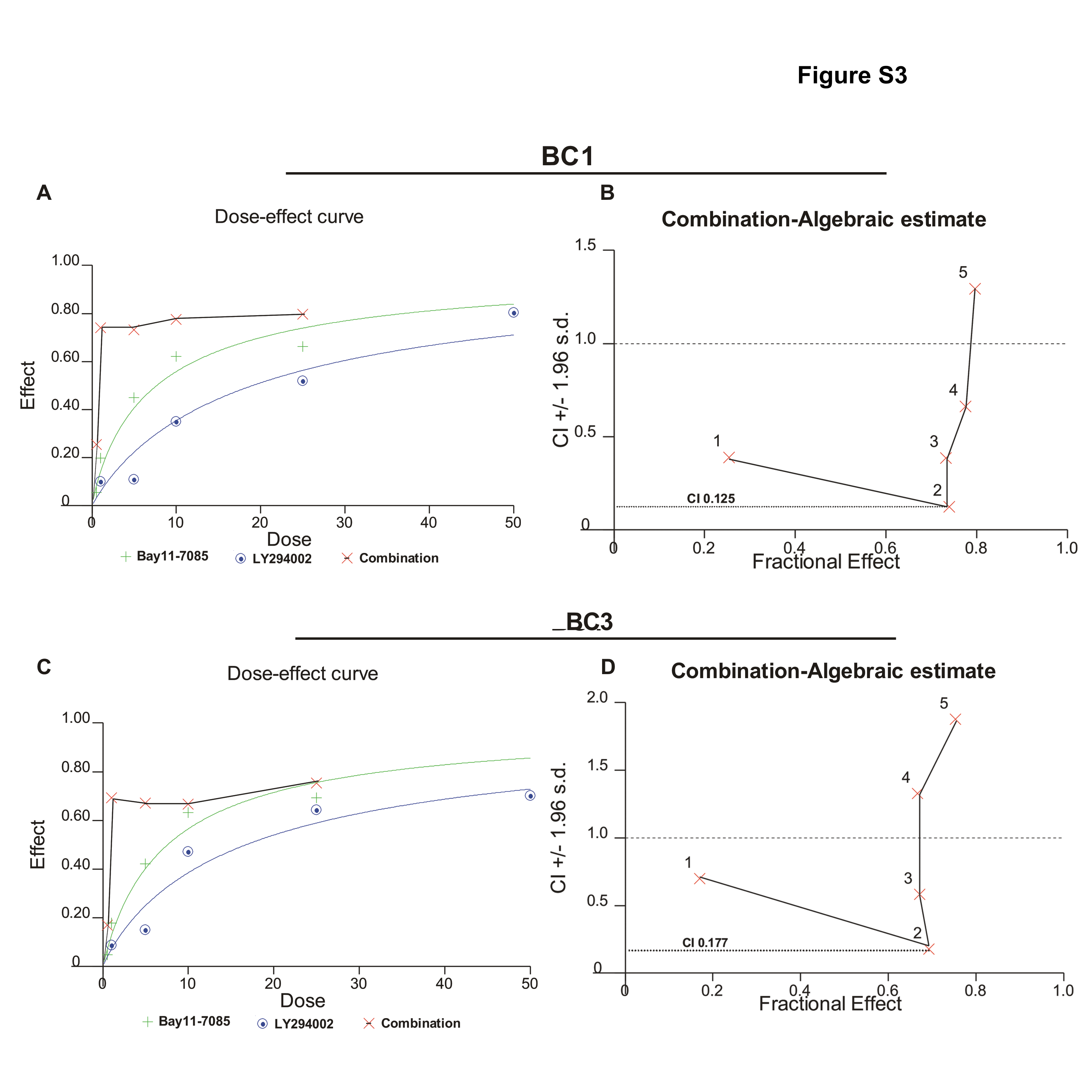

Supplement: Figure S3 — Synergistic apoptotic response of Bay11-7085 and TRAIL in PEL cells. BC1 and BC3 cells were treated with various combinations of Bay11-7085 and TRAIL for 24 hours and dose effect (A and C) and Fractional effect (B and D) graphs were generated using Calcusyn software. Apoptotic response analysis was measured as mean ± SD values normalized to control. Combination indices were calculated using Chou and Talalay methodology. (TIF) [file pone.0039945.s003.tif]
