# Supplementary figures and images for: Correction: Cross-Talk between NFkB and the PI3-Kinase/AKT Pathway Can Be Targeted in Primary Effusion Lymphoma (PEL) Cell Lines for Efficient Apoptosis
Source: PLoS One. 2014 Mar 10;9(3):e92484. doi: 10.1371/journal.pone.0092484 (PMC3948945; doi:10.1371/journal.pone.0092484)

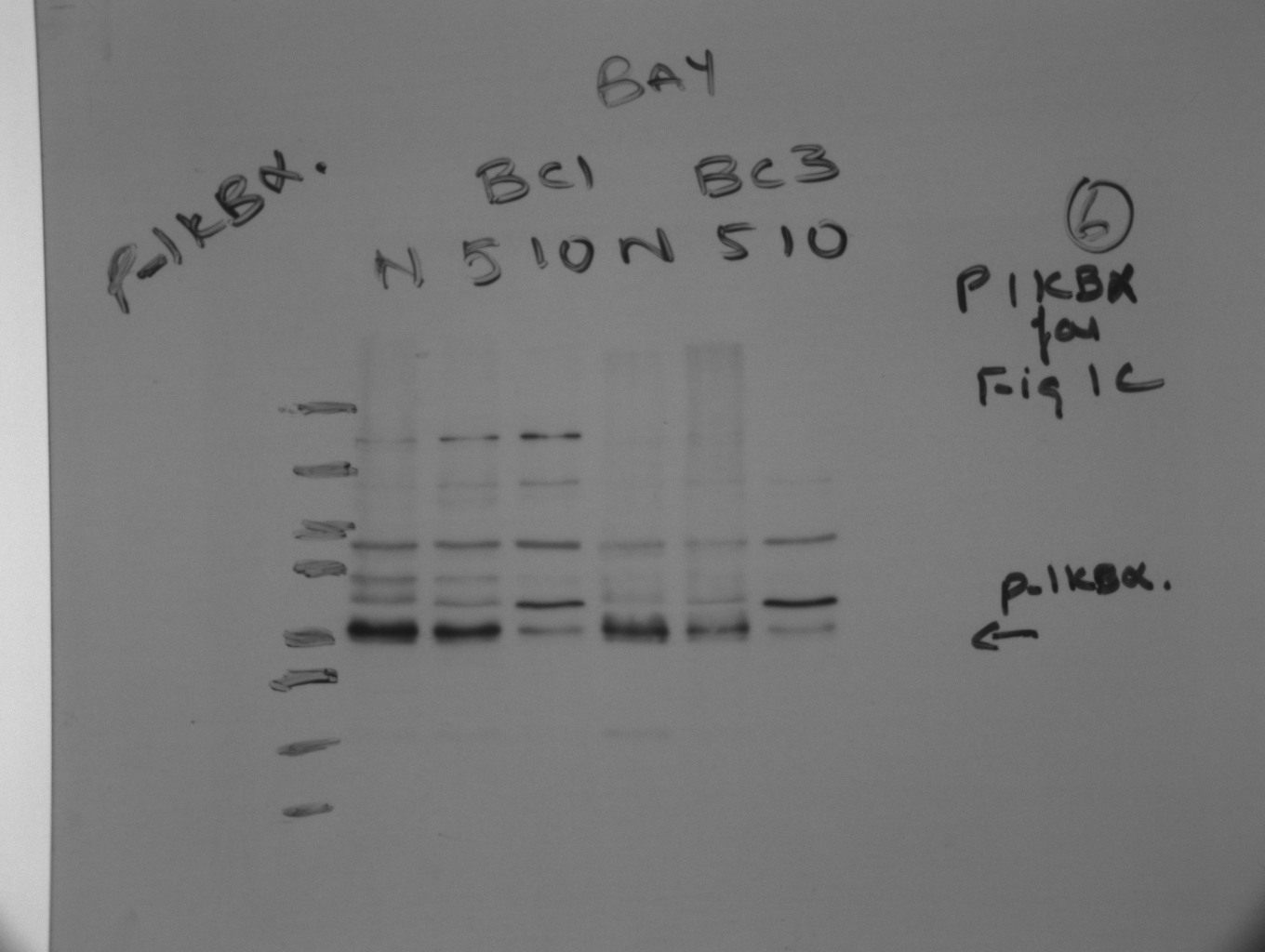

Supplement: File S1 — Raw blots for Figure 1C (ZIP) [file pone.0092484.s001.zip › 1. Raw blot for p-IKappaBAlpha for Figure 1C.tif]

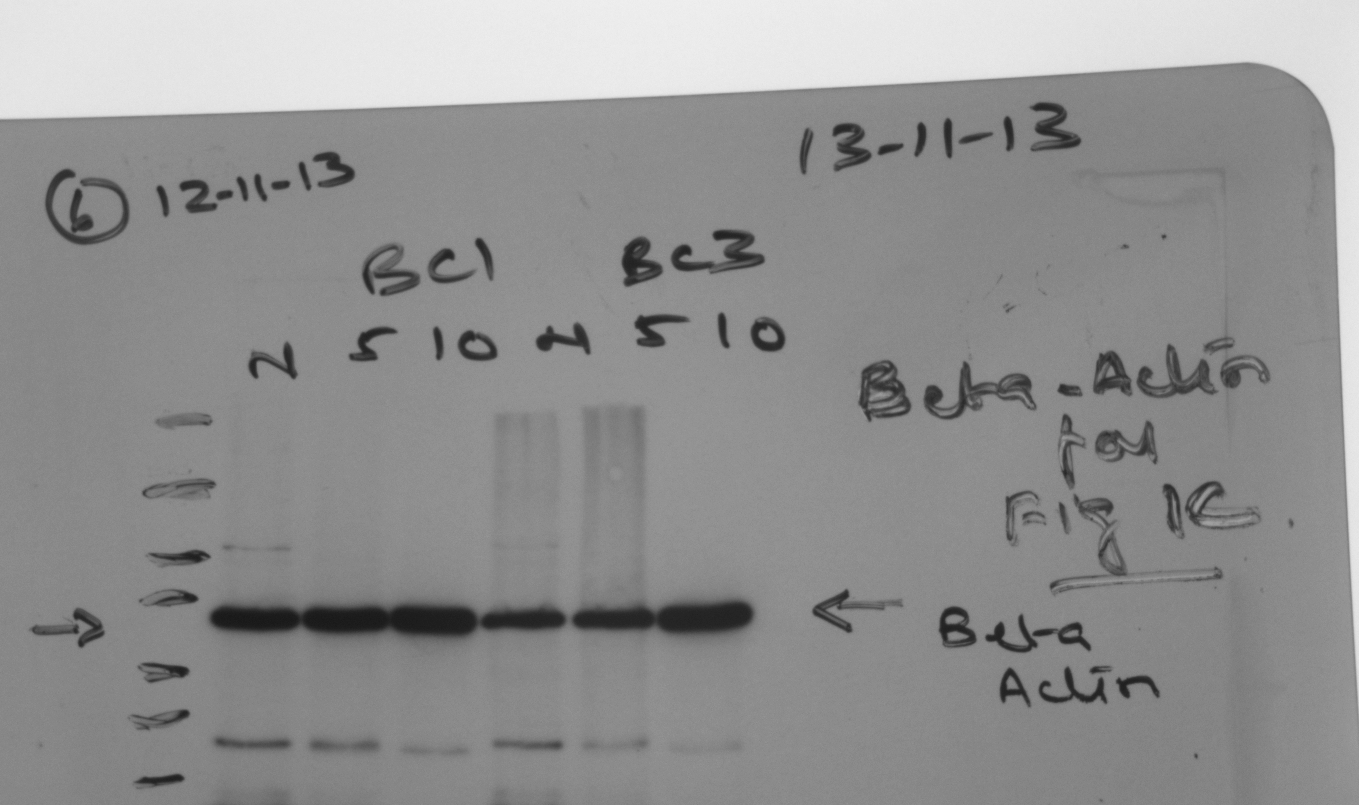

Supplement: File S1 — Raw blots for Figure 1C (ZIP) [file pone.0092484.s001.zip › 2. Raw blot for Beta-Actin for Figure 1C.tif]

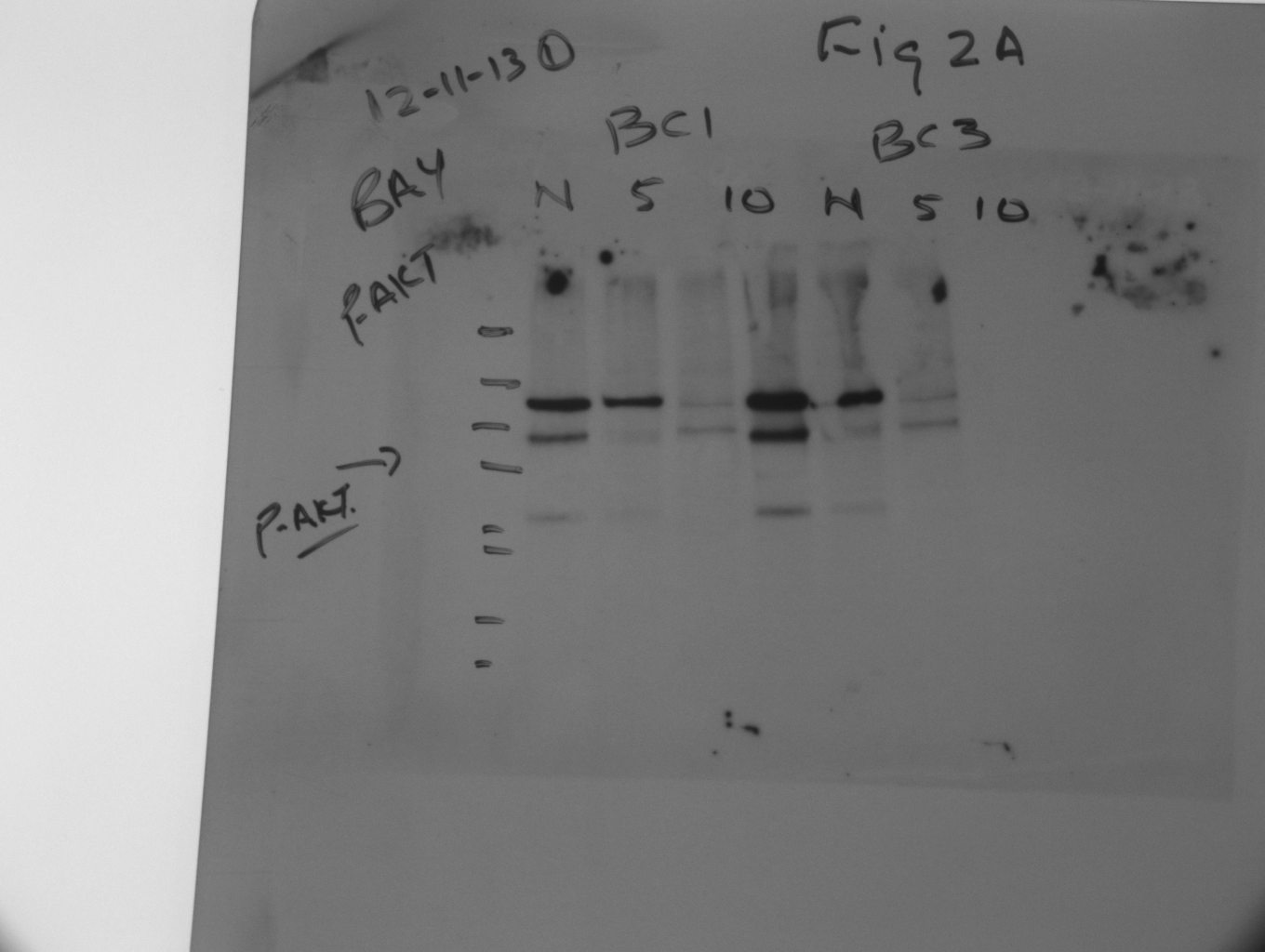

Supplement: File S2 — Raw blots for Figure 2A (ZIP) [file pone.0092484.s002.zip › 1. Raw blot for p-AKT for Fig 2A.tif]

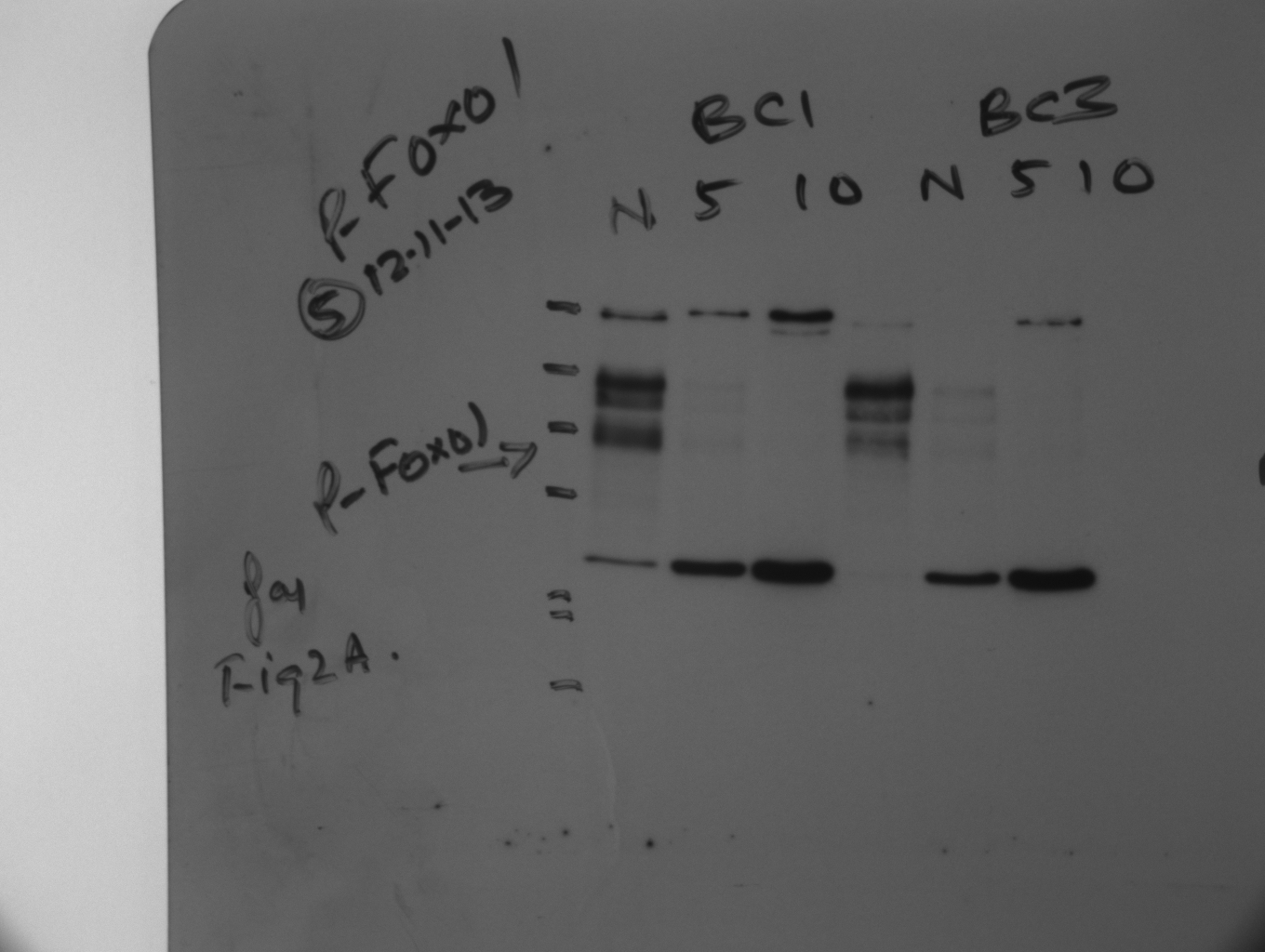

Supplement: File S2 — Raw blots for Figure 2A (ZIP) [file pone.0092484.s002.zip › 2. Raw blot for p-FOXO1 for Fig 2A-2.tif]

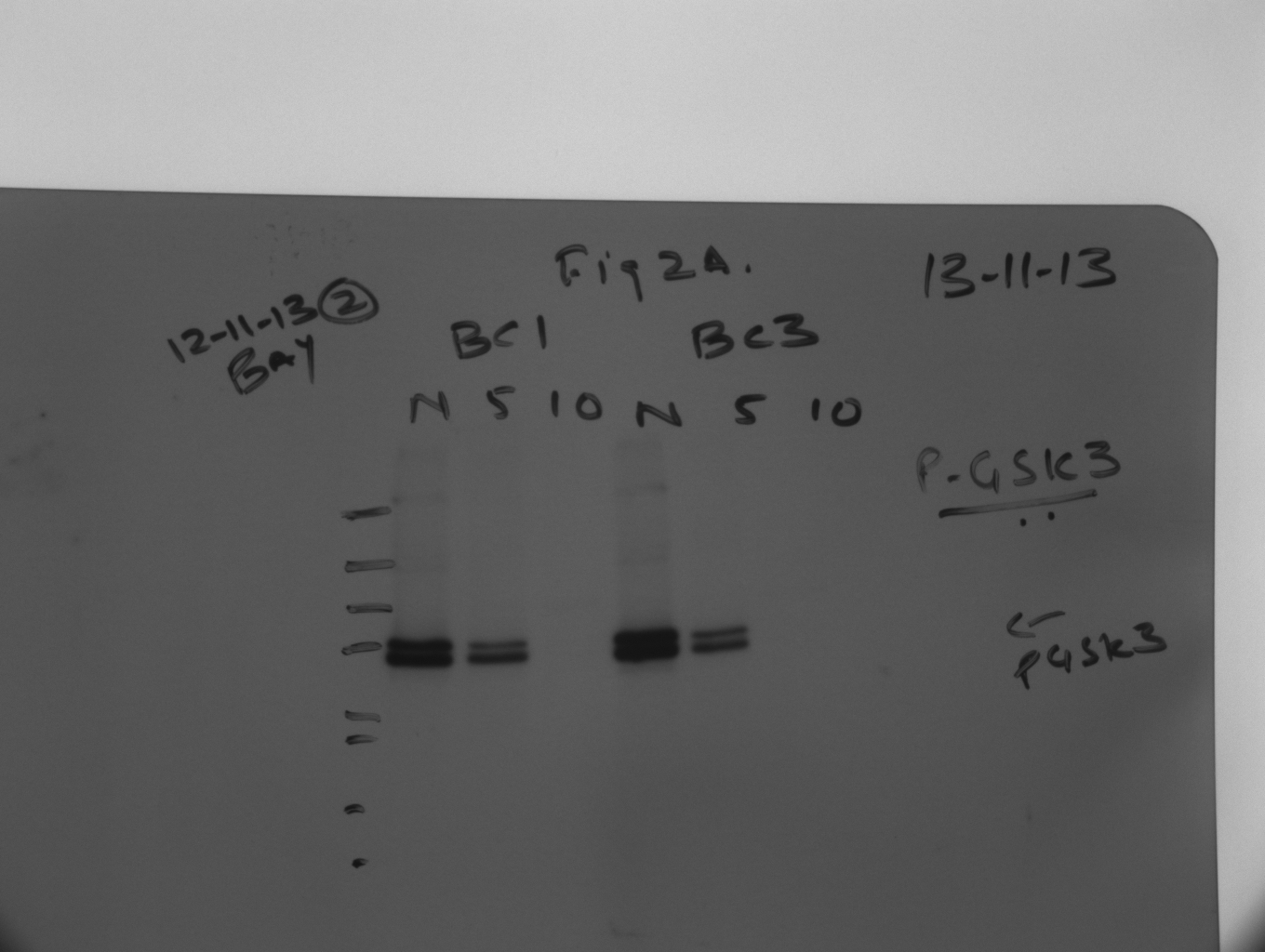

Supplement: File S2 — Raw blots for Figure 2A (ZIP) [file pone.0092484.s002.zip › 3. Raw blot for p-GSK3 for Fig 2A.tif]

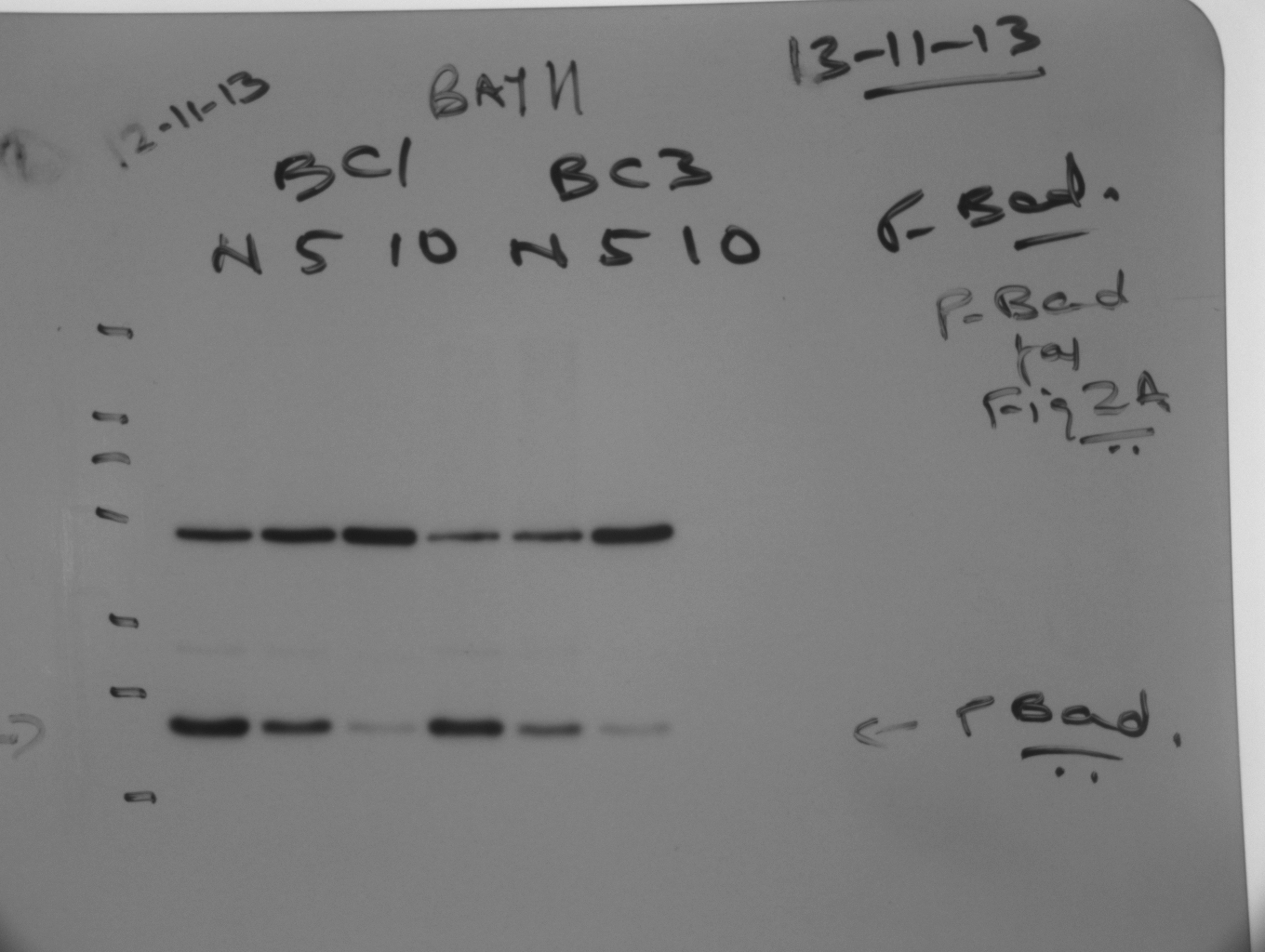

Supplement: File S2 — Raw blots for Figure 2A (ZIP) [file pone.0092484.s002.zip › 4. Raw blot for p-Bad for Fig 2A.tif]

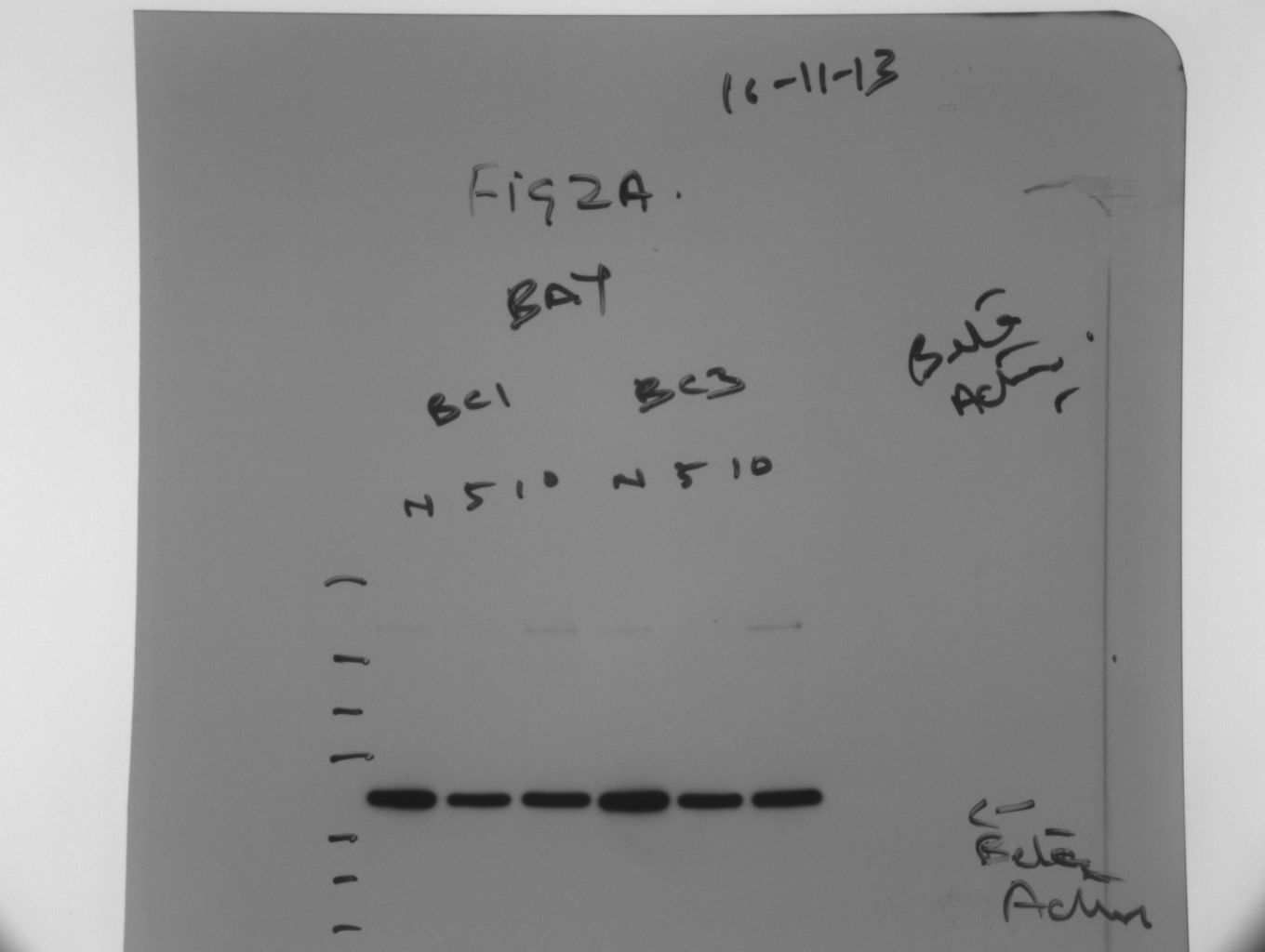

Supplement: File S2 — Raw blots for Figure 2A (ZIP) [file pone.0092484.s002.zip › 5. Raw blot for Beta-Actin for Fig 2A Raw data.tif]

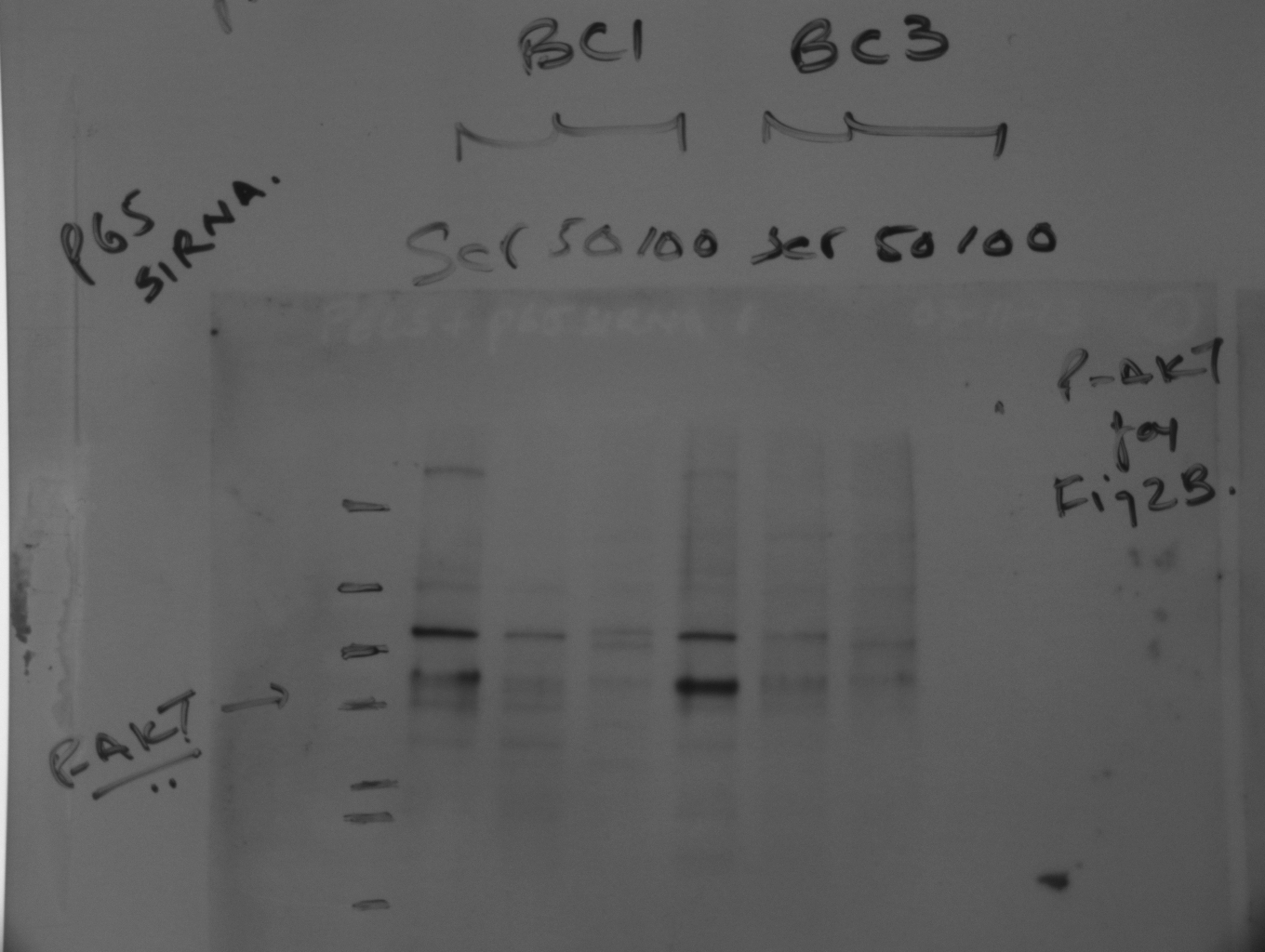

Supplement: File S3 — Raw blots for Figure 2B (ZIP) [file pone.0092484.s003.zip › 1. Raw blot for p-AKT for Figure 2B.tif]

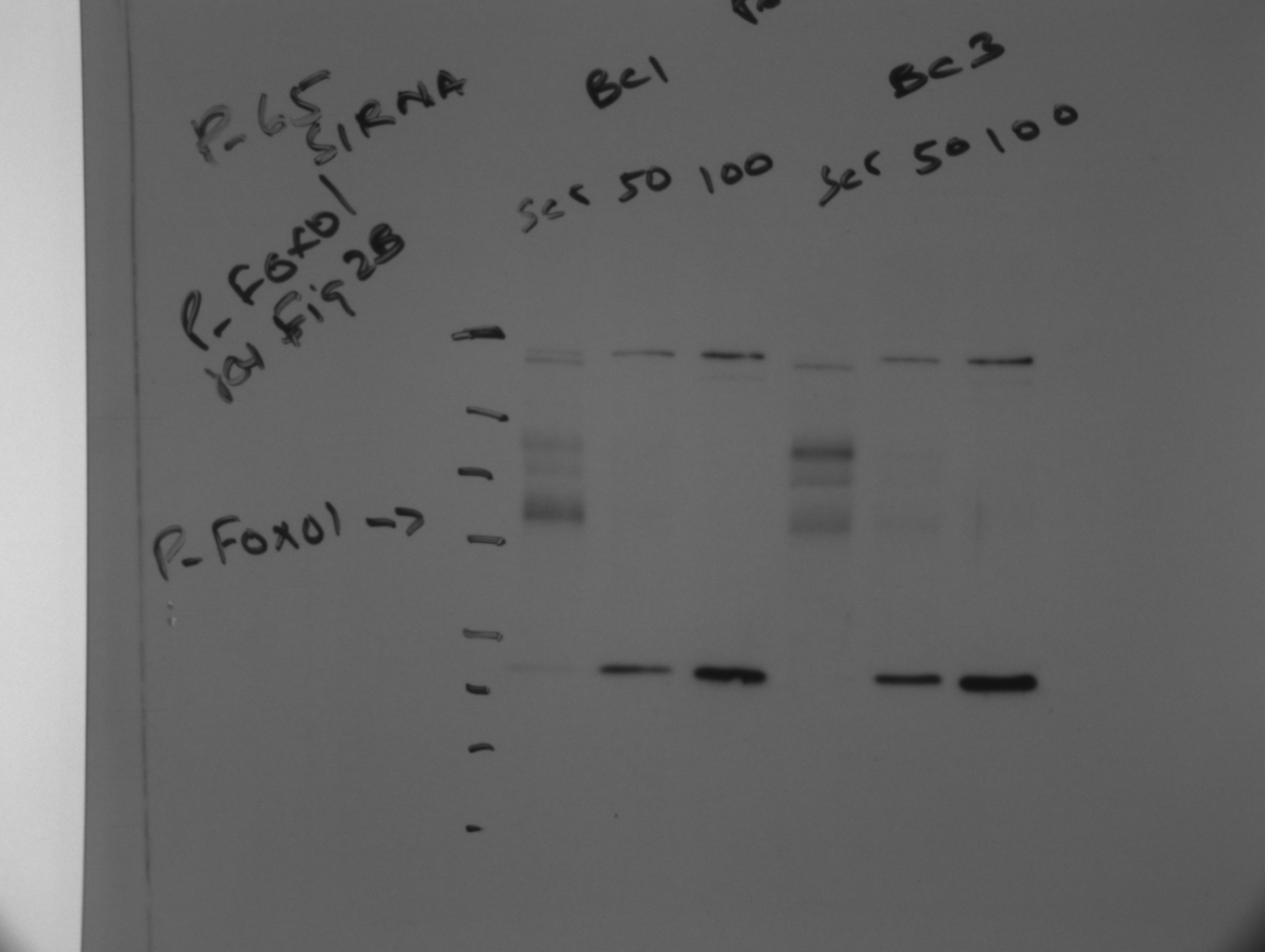

Supplement: File S3 — Raw blots for Figure 2B (ZIP) [file pone.0092484.s003.zip › 2. Raw blot for p-FOXO1 for Figure 2B.tif]

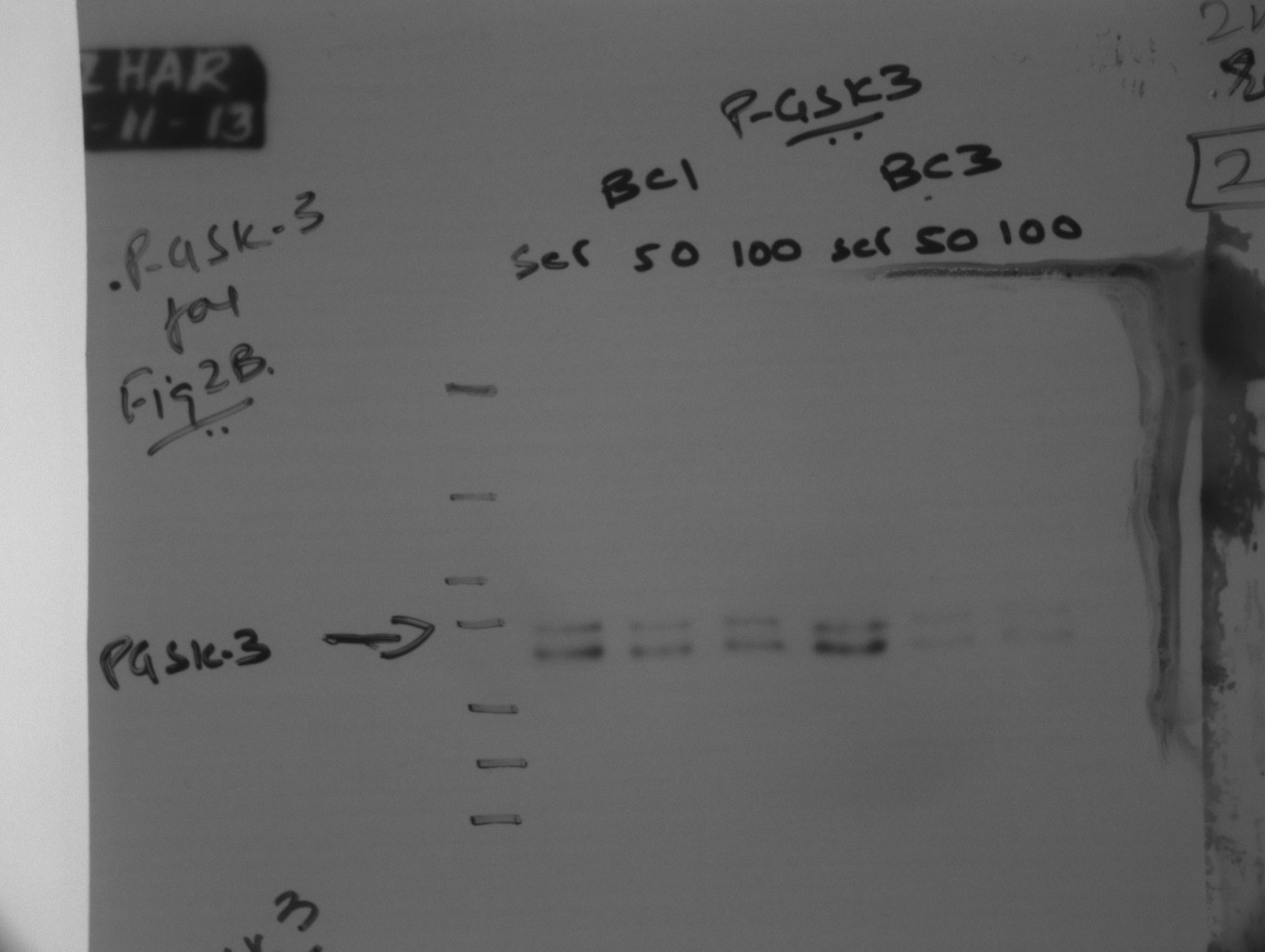

Supplement: File S3 — Raw blots for Figure 2B (ZIP) [file pone.0092484.s003.zip › 3. Raw blot for p-GSK3 for Figure 2B.tif]

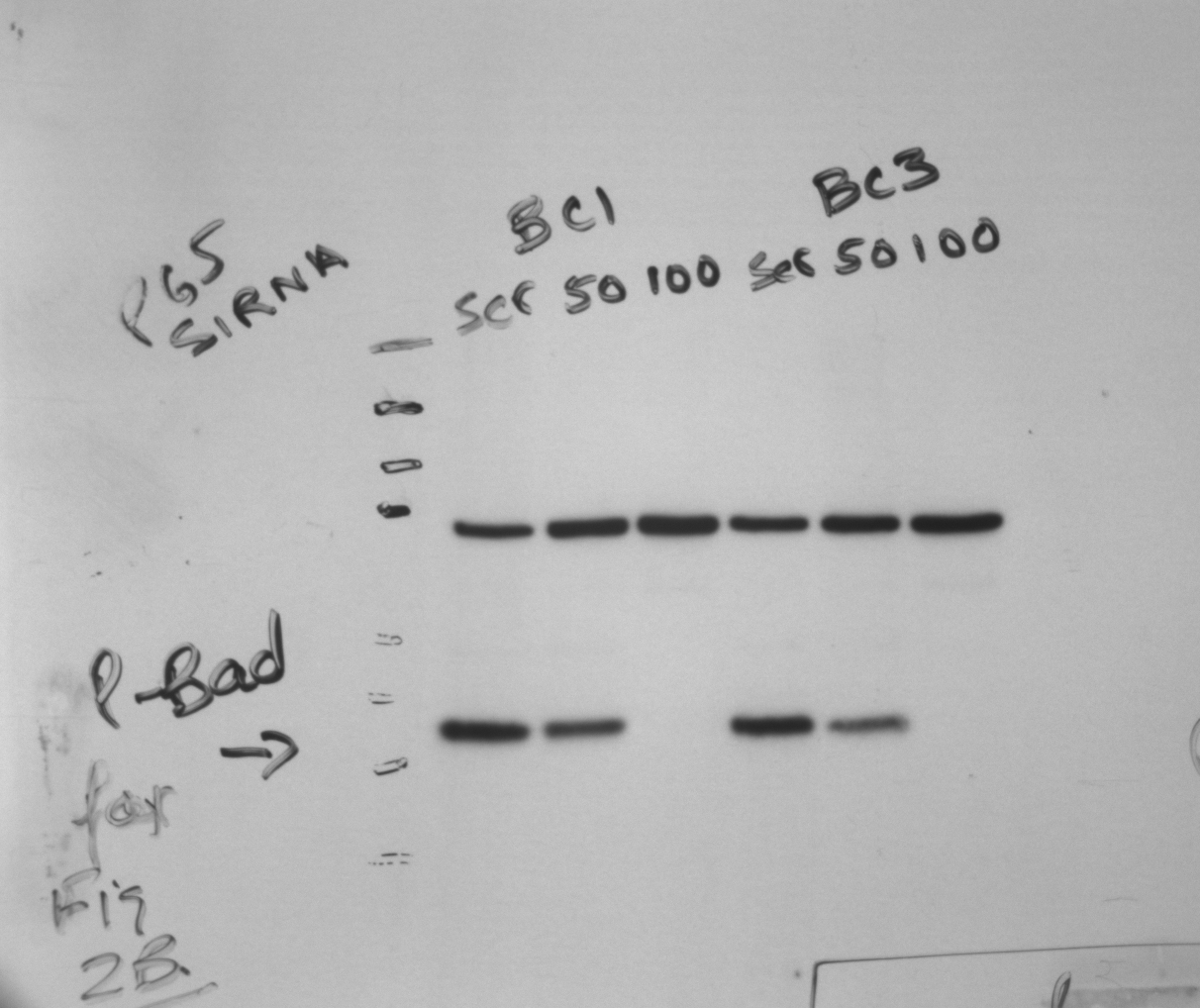

Supplement: File S3 — Raw blots for Figure 2B (ZIP) [file pone.0092484.s003.zip › 4. Raw blot for p-Bad for Figure 2B.tif]

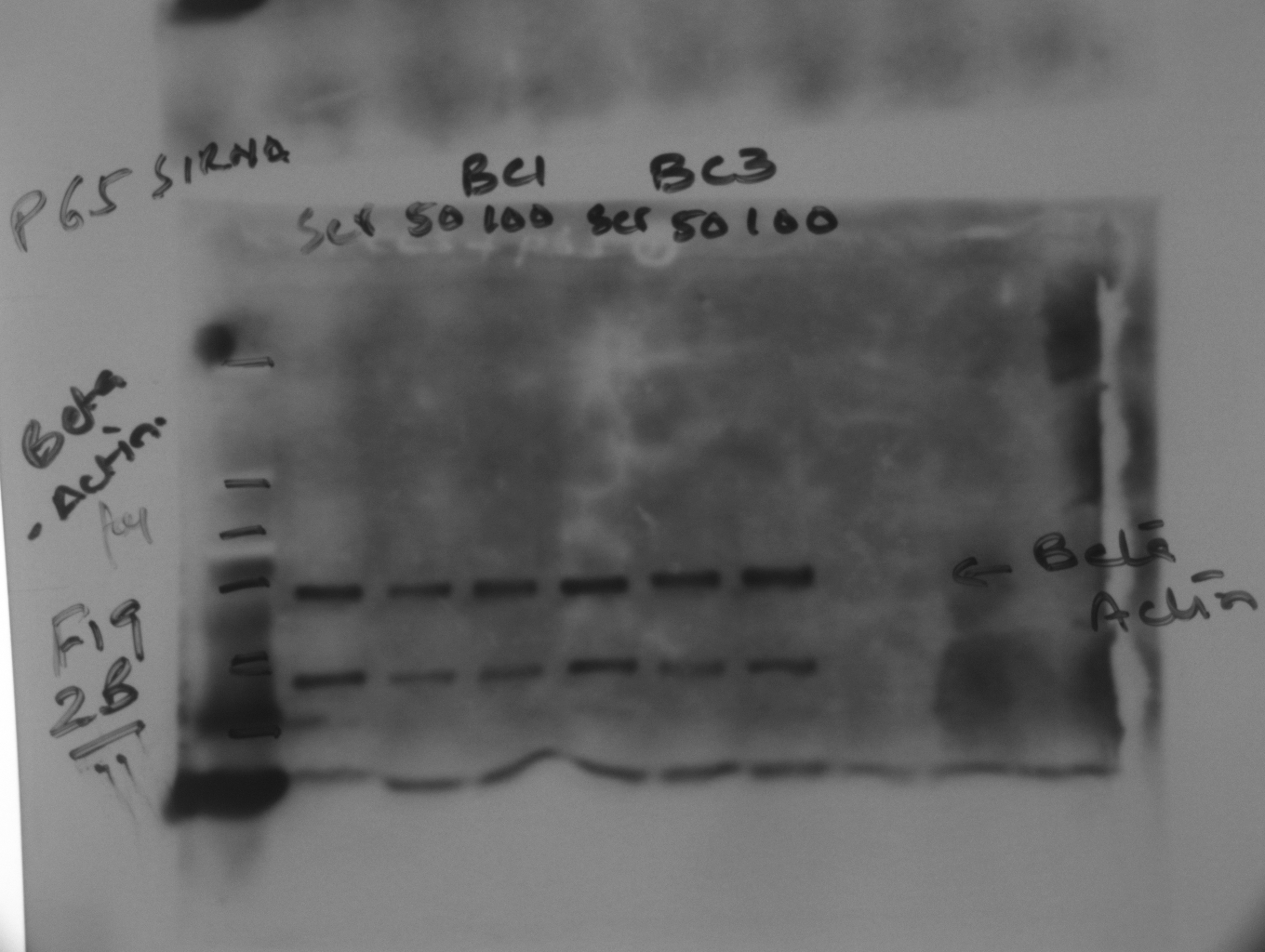

Supplement: File S3 — Raw blots for Figure 2B (ZIP) [file pone.0092484.s003.zip › 5. Raw blot for Beta-Actin For Figure 2B.tif]

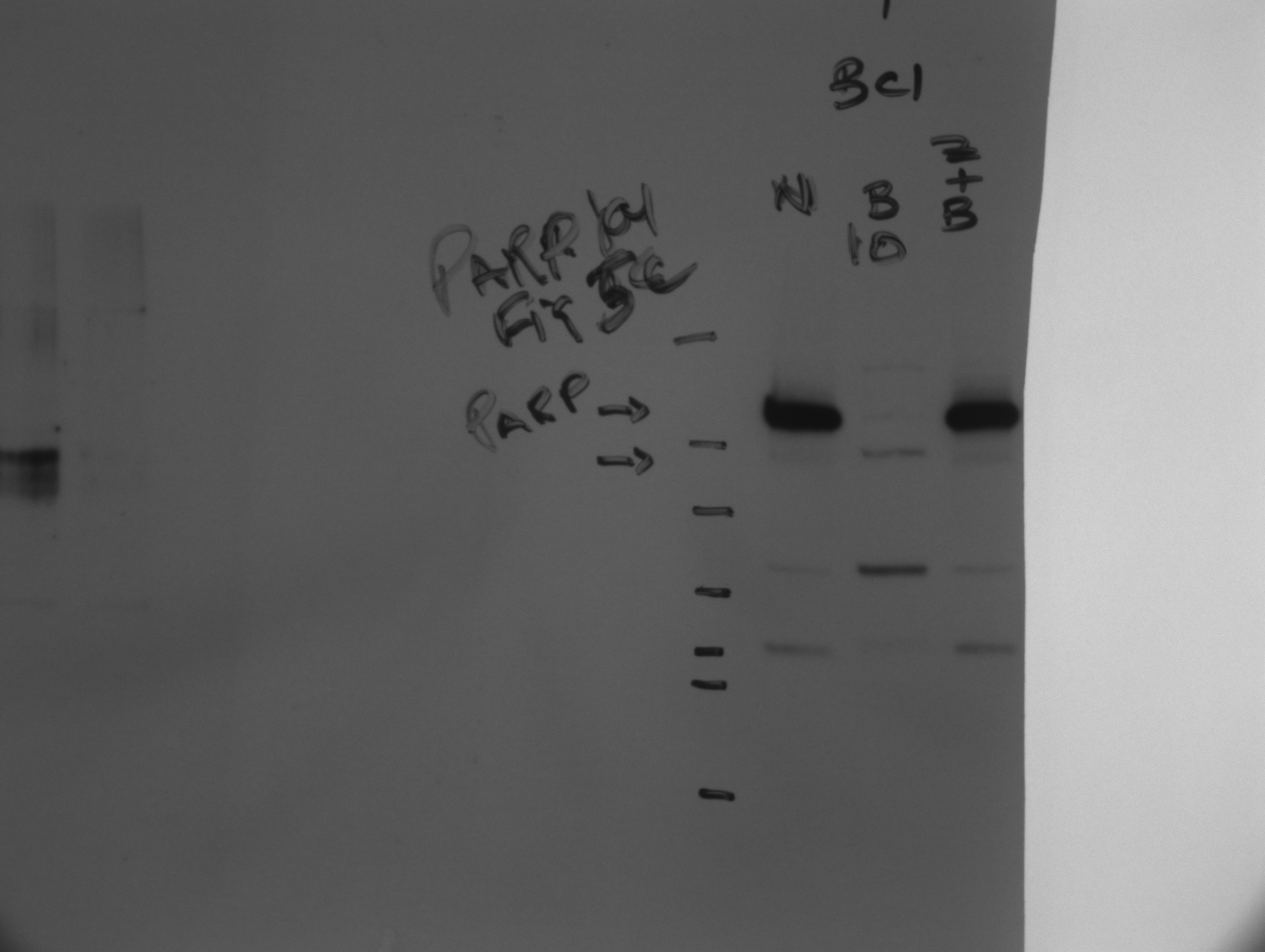

Supplement: File S4 — Raw blots for Figure 5C (ZIP) [file pone.0092484.s004.zip › 1. Raw blot for PARP For Figure 5C.tif]

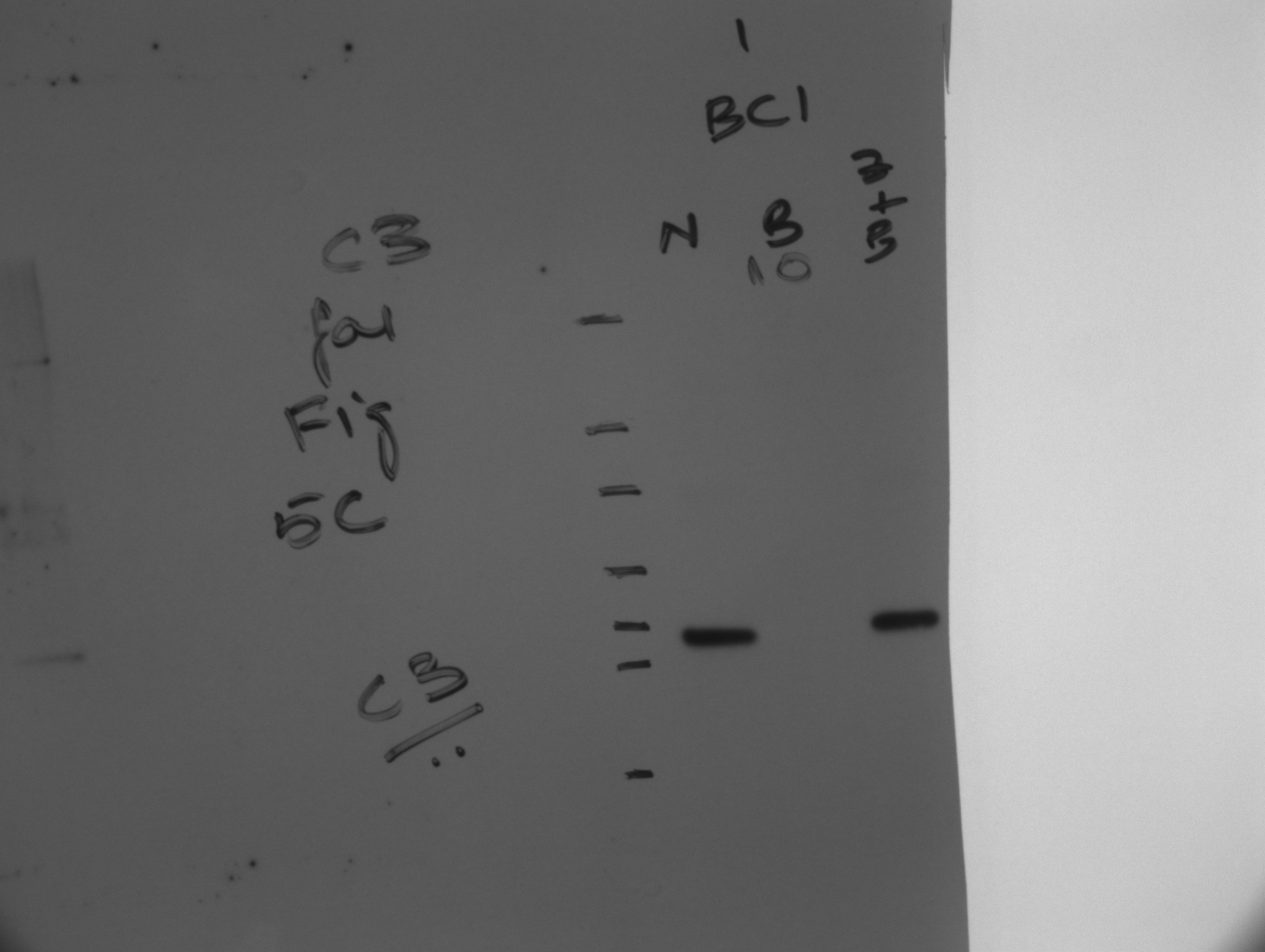

Supplement: File S4 — Raw blots for Figure 5C (ZIP) [file pone.0092484.s004.zip › 2. Raw blot for Caspase-3 For Figure 5C.tif]

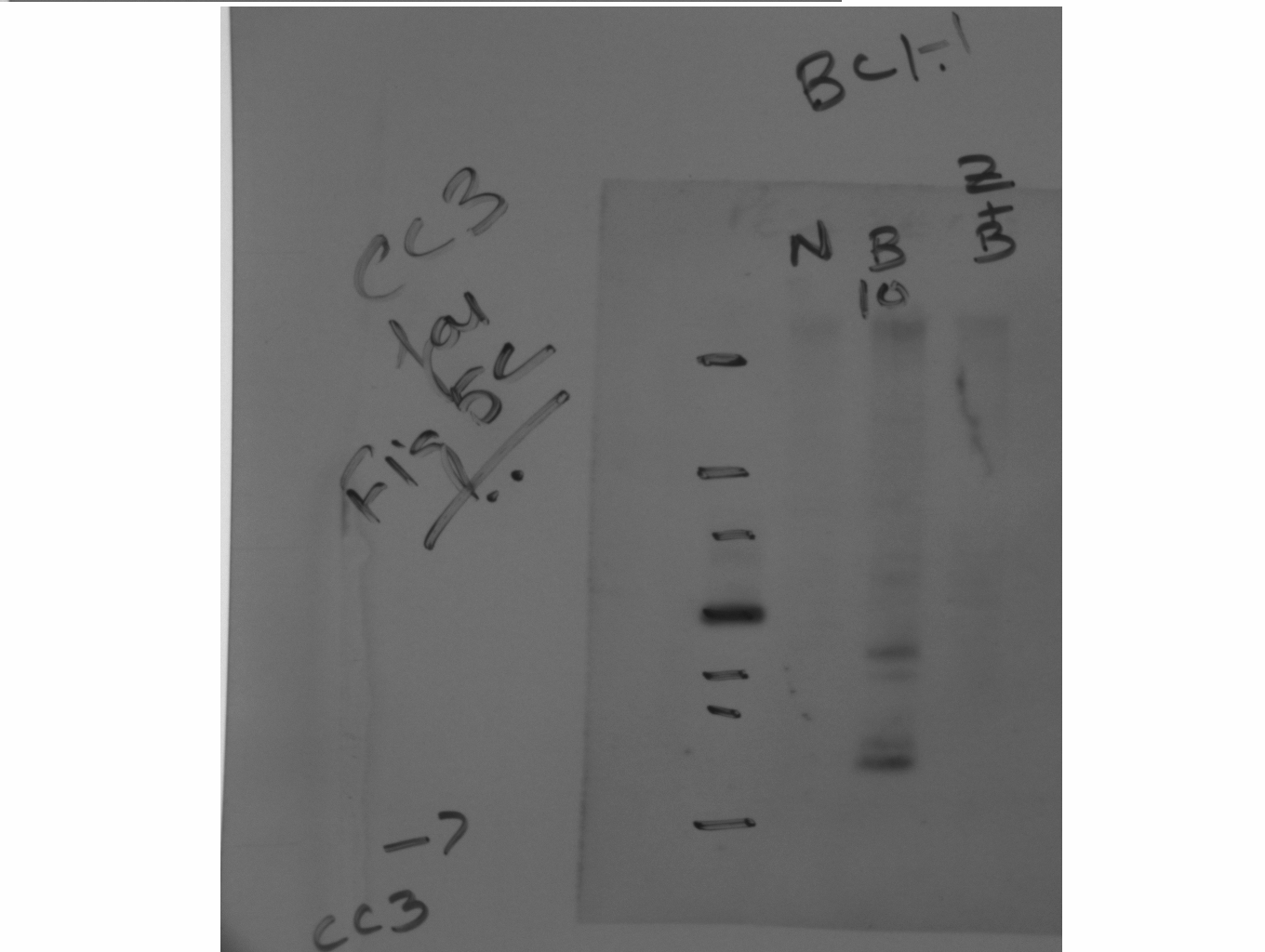

Supplement: File S4 — Raw blots for Figure 5C (ZIP) [file pone.0092484.s004.zip › 3. Raw blot for Cleaved caspase-3 For Figure 5C.tif]

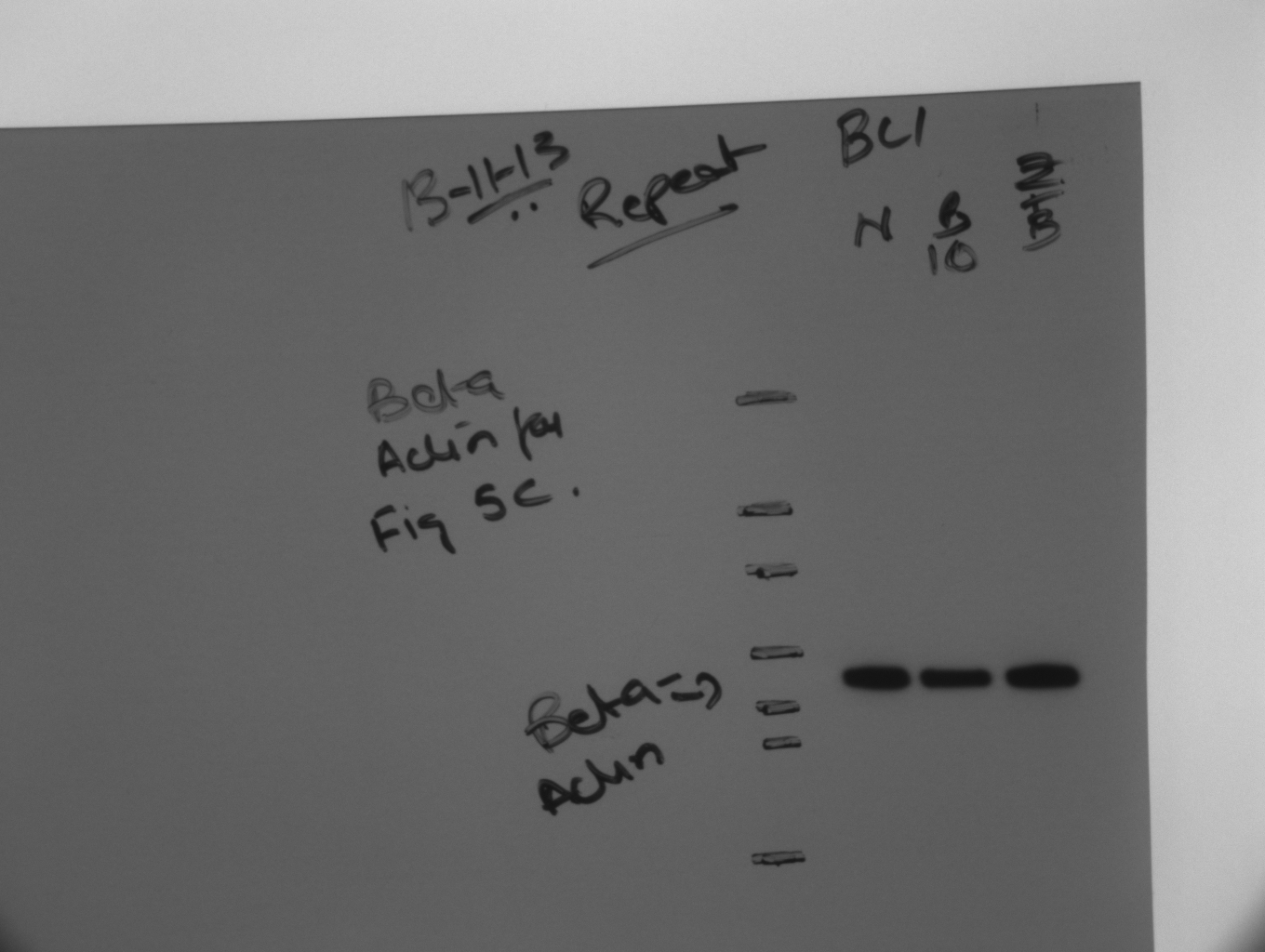

Supplement: File S4 — Raw blots for Figure 5C (ZIP) [file pone.0092484.s004.zip › 4. Raw blot for Beta-Actin For Figure 5C.tif]
